# Tumor cell-intrinsic BIN1 deficiency promotes the immunosuppression and impedes ferroptosis of non-small cell lung cancer via G3BP1-mediated degradation of STAT1

**DOI:** 10.1186/s13046-025-03404-9

**Published:** 2025-05-09

**Authors:** Jiali Wang, Yunlong Jia, Tianxu Liu, Xinyan Liu, Shuxian Yin, Jiaqi Chen, Xiaoqing Xu, Yi Zhang, Lihua Liu

**Affiliations:** 1 Department of Tumor Immunotherapy, Hebei Medical University Fourth Affiliated Hospital and Hebei Provincial Tumor Hospital, Shijiazhuang, 050035 P.R. China; 2 Department of General Surgery, Xuanwu Hospital Capital Medical University, Beijing, 100053 China; 3 Biotherapy Center and Cancer Center, The First Affiliated Hospital of Zhengzhou University, Zhengzhou, Henan 450002 China; 4 China International Cooperation Laboratory of Stem Cell Research, Hebei Medical University, Shijiazhuang, 050011 China

**Keywords:** BIN1, Non-small cell lung cancer, CD8^+^ T cell, Immune escape, Ferroptosis

## Abstract

**Background:**

Tumors often evade immune surveillance by limiting T cell infiltration. In non-small cell lung cancer (NSCLC), increased infiltration of CD8^+^ T cells is associated with a favorable response to immunotherapy. While BIN1 is recognized as a tumor suppressor gene, its role in shaping the tumor microenvironment in NSCLC has yet to be fully clarified.

**Methods:**

To investigate the relationship between BIN1 expression and CD8^+^T cell infiltration in NSCLC, we performed a comprehensive data analysis utilizing clinical information from NSCLC patients. BIN1 expression levels in NSCLC tissues were evaluated, and their correlation with CD8^+^T cells infiltration and patient survival outcomes was examined. Loss-of-function strategies targeting BIN1 were applied in syngeneic NSCLC mouse models to assess its functional significance. Tumor growth was monitored, and immune cell populations were analyzed in terms of frequency and functionality through mass cytometry and flow cytometry techniques. Cytokine secretion was profiled using multiplex assays. Additionally, RNA sequencing, immunoprecipitation-mass spectrometry, and molecular docking were employed to confirm direct interactions between BIN1 and cytokine-encoding genes. Finally, the regulatory role of BIN1 in ferroptosis in NSCLC cells were explored using metabolomics analysis, ROS measurement, and MDA detection.

**Results:**

We observed that BIN1 expression is downregulated in NSCLC tumor tissues, with its reduced expression strongly associated with advanced disease progression and poor prognosis. Bioinformatics analysis of immune infiltration in human NSCLC samples revealed a positive correlation between BIN1 expression in NSCLC tissues and CD8^+^ T cell infiltration. Furthermore, the prognostic impact of BIN1 on NSCLC patients is strongly linked to the level of CD8^+^ T cell infiltration. In syngeneic mouse models, the knockout of BIN1 in NSCLC cells significantly inhibited CD8^+^ T cell infiltration and impaired their cytotoxic function, facilitating tumor immune evasion. Mechanistically, we demonstrated that BIN1 directly interacts with G3BP1, and its knockout stabilizes G3BP1. This, in turn, promotes STAT1 degradation and reduces the secretion of T cell-recruiting chemokines such as CXCL10 and CCL5. Finally, our findings reveal that BIN1 influences ferroptosis in NSCLC cells through the G3BP1/STAT1/GSH pathway, thereby regulating NSCLC cell proliferation, migration, and invasion.

**Conclusion:**

This study highlights the crucial role of the BIN1/G3BP1/STAT1/CD8^+^ tumor-infiltrating lymphocyte signaling pathway in the progression of NSCLC and its mechanisms of immune evasion. This fundings lay a foundation for the development of BIN1-targeted therapies aimed at improving tumor immunogenicity and transforming immunologically “cold” NSCLC into a more responsive disease.

**Supplementary Information:**

The online version contains supplementary material available at 10.1186/s13046-025-03404-9.

## Background

Recent advancements in the treatment of metastatic non-small cell lung cancer (NSCLC) have been realized through the introduction of targeted therapies, such as EGFR inhibitors for EGFR-mutated NSCLC, and immunotherapies, including anti-programmed cell death protein 1 (PD-1) antibodies [1–4]. However, resistance to targeted therapies inevitably emerges over time. While some patients achieve durable responses with anti-PD-1 antibody therapy, approximately 70–80% either fail to respond effectively or develop resistance to these treatments [5–6]. Consequently, a comprehensive understanding of the cellular and molecular mechanisms driving phenotypic adaptations is essential to uncover the remarkable ability of NSCLC cells to resist current therapeutic approaches. Such insights are critical for advancing the development of novel combination therapies.

The immunosuppressive tumor microenvironment (TME) plays a pivotal role in tumor immune evasion and resistance to immunotherapy [7], as demonstrated by the exhaustion and diminished infiltration of cytotoxic tumor-infiltrating lymphocytes (TILs), particularly CD8^+^ T cells [8–10]. CD8^+^ T cells possess the unique ability to selectively identify and eliminate cancer cells. Tumors express antigens, which include tumor-specific (mutant and viral) neoantigens and self-antigens (also referred to as tumor-associated or shared antigens). Notably, CD8^+^ T cells reactive to these antigens have been detected in cancer patients [11]. Interestingly, both the presence and the differentiation and localization of TILs have been shown to significantly influence clinical outcomes [12–15]. While numerous studies have investigated the immune profiles and heterogeneity of the NSCLC TME using mouse models and human patient samples, the exact mechanisms of immune evasion involving CD8^+^ T cells remain inadequately understood. A deeper understanding of these mechanisms could unveil predictive biomarkers and identify potential drug targets, ultimately guiding the development of more effective immunotherapeutic strategies.

The loss of the BIN1 tumor suppressor is among the most common oncogenic drivers across various cancers, including esophageal, gastric, neuroblastoma, breast, lung, colorectal, prostate, pancreatic cancers, and malignant pleural mesothelioma [16, 17]. Mounting evidence underscores the strong association between aberrant BIN1 expression and cancer development. For instance, reduced BIN1 expression collaborates with RAS activation to promote tumor progression in breast cancer, highlighting BIN1’s role as a negative regulator of breast cancer carcinogenesis and progression [18]. BIN1 also plays a crucial role in inhibiting immune evasion in cancer cells by suppressing the STAT1 and NF-κB pathways, which in turn reduces the expression of the immunoregulatory enzyme IDO [19]. However, this effect is significantly impaired in BIN1-deficient cells. Our prior research revealed that BIN1 regulates the expression of PD-L1 in tumor cells and that its expression is strongly associated with CD8^+^ T cell infiltration in NSCLC [20]. Nevertheless, the mechanisms underlying the downregulation of endogenous BIN1 expression during oncogenic transformation remain intricate and are not yet fully understood.

We demonstrated that BIN1 ablation suppresses CD8^+^ T cell infiltration in NSCLC mouse models through mass cytometry, multicolor flow cytometry, and multicolor immunofluorescence staining. Using immunoprecipitation-mass spectrometry, RNA sequencing (RNA-seq), and molecular docking analyses, we further revealed that reduced BIN1 expression inhibits the activation of signal transducer and activator of transcription 1 (STAT1) and STAT1-mediated transcription of CXCL10 and CCL5 by stabilizing G3BP1 protein. This process ultimately diminishes tumor-infiltrating T cells. Additionally, through metabolomics and complementary experimental approaches, we discovered that BIN1 knockout leads to glutathione (GSH) accumulation and suppresses ferroptosis in NSCLC cells via the G3BP1/STAT1 pathway, thus promoting NSCLC progression. These findings provide novel mechanistic insights into the role of BIN1 in tumor immune evasion and the advancement of NSCLC.

## Materials and methods

### Cell culture and reagents

The mouse NSCLC cell line, LLC, was procured from the American Type Culture Collection (Manassas, Virginia, USA). All cells were maintained in high-glucose Dulbecco’s Modified Eagle’s Medium (DMEM) (Gibco BRL, Grand Island, New York, USA), supplemented with 10% (v/v) fetal bovine serum and 1% penicillin/streptomycin. Cultures were incubated at 37 °C in a humidified atmosphere containing 5% CO₂.

The STAT1 agonist SB02024 (#HY-122891), ferroptosis inducer erastin (#HY-15763), ferroptosis inhibitor ferrostatin-1 (HY-100579), pan caspase inhibitor Z-VAD (#HY-164388), and autophagy inhibitor 3-Methyladenine (3-MA) (#HY-19312) were procured from MedChemExpress.

### SgRNA infection

Design the sgRNA sequences targeting gene BIN1 using online tools, such as the CRISPR Design Tool. The designed sequences are as follows: sgRNA-1: 5’-CAGCTTCTTCTGTACGTTGC-3’. sgRNA-2: 5’-GAAGGATCTTCGGACCTATC-3’. sgRNA-3: 5’-ACACTCACTCAGCTTCTTGG-3’. Synthesize the selected sgRNA and confirm its sequence accuracy. Construct the editing vector by cloning the sgRNA sequence into a plasmid vector capable of expressing the Cas9 protein. Perform bacterial transformation and subsequent plasmid extraction to obtain the recombinant plasmid.

Cell culture and transfection: Cultivate LLC cells under appropriate conditions. Employ liposome-based transfection reagents to introduce the recombinant plasmid into the cultured cells.

Gene knockout screening and verification: Select successfully transfected cells using antibiotics and confirm the gene knockout effect through Western blot analysis and Sanger sequencing.

### Lentiviral infection

Lentiviruses containing short hairpin RNAs (shRNA) targeting G3BP1 were obtained from Shanghai HANBIO Co., Ltd (mm-G3BP1-sh-1:5’-GAUGAGGUCUUCGGUGGCUUUdTdT-3’. mm-G3BP1-sh-2:5’-GGAACUUUCU AUGAUCAGAdTdT-3’. mm-G3BP1-sh-3:5’-GAGAGCAGCGAAUCAAUAUdTdT-3’). These lentiviruses, designed to deliver knockdown elements, were used to infect LLC cells following the manufacturer’s guidelines. Infected cells were subsequently selected for using puromycin.

### Cell proliferation, migration, and invasion assay analysis

Cell viability was assessed using the EdU assay (#C0071S, Beyotime Biotechnology) following the manufacturer’s instructions.

Wound-healing migration was evaluated using a specialized assay. Photographs were captured at 0, 24, and 48 h, and the scratched area was measured using ImageJ software.

Transwell migration assays were conducted using cell culture inserts. Genetically modified cells (2 × 10⁵ cells/mL) in FBS-free DMEM were prepared, and 200 µL of differently treated LLC cells was washed with PBS and seeded into the top chamber. Meanwhile, 600 µL of DMEM supplemented with 20% FBS was added to the bottom chamber. Following a 24-hour incubation, the cell culture inserts were fixed with 1% paraformaldehyde for 20 min and subsequently stained with 0.1% crystal violet for 15 min. Non-migrating cells were carefully removed using a swab. The average number of stained cells per field was quantified by examining four separate fields under 100× magnification.

Cell invasion was evaluated using a Matrigel-coated Transwell invasion assay. A layer of Matrigel (200 mg/mL) was pre-applied to the upper chamber on ice to create a mimetic extracellular matrix barrier. The remaining steps were carried out in accordance with the protocol for the migration assay.

### Western blotting

Proteins were extracted using RIPA lysis buffer (#P0013C, Beyotime Biotechnology) and quantified with a BCA detection kit (#P0012, Beyotime Biotechnology) according to the manufacturer’s instructions. Equal amounts of protein were resolved by SDS-PAGE, transferred onto a polyvinylidene difluoride (PVDF) membrane via electrophoresis, and blocked with 5% nonfat milk in Tris-buffered saline for 1 h at room temperature. The membrane was then incubated overnight at 4 °C with specific primary antibodies (BIN1, 1:5000; G3BP1, 1:5000; STAT1, 1:4000; GAPDH, 1:10,000), followed by thorough washing and subsequent incubation with secondary antibodies for 1 h at room temperature. Protein bands were visualized using an enhanced chemiluminescence reagent (#P0018S, Beyotime Biotechnology).

### Immunohistochemistry

Specimens were embedded in paraffin, and serial sections, 4 μm thick, were prepared. The sections were subsequently deparaffinized, blocked, and incubated overnight at 4 °C with primary antibodies, including Ki67 (1:200), BIN1 (1:100), G3BP1 (1:50), and STAT1 (1:100). This was followed by incubation with a horseradish peroxidase-labeled secondary antibody. Human lung cancer tissue microarrays (RLN186) were obtained from Shanghai Zhuoli Biotech Co., Ltd, comprising 70 lung cancer tissue samples (63 with NSCLC and 7 with SCLC) and 10 normal lung tissue samples. The H-Score of BIN1 and CD8 expression was calculated through the IHC-profilter plugin in Imagine J. Clinical and pathological data for the samples were provided by the array manufacturer.

### RNA isolation and quantitative real-time PCR

Total RNA was extracted using MolPure^®^ TRIeasy™ Plus Total RNA Kit (#19211ES60, Yeasen) and reverse-transcribed using Hifair^®^ AdvanceFast 1st Strand cDNA Synthesis SuperMix for qPCR (#1156ES60, Yeasen). Quantitative real-time PCR was performed using Hieff^®^ qPCR SYBR Green Master Mix (No Rox) (#11201ES08, Yeasen) on a real-time PCR system (LightCycler 96). Expression was normalized to GAPDH by the 2^–ΔΔ^Ct method.

### Multicolor cytometry

Immune cell populations were analyzed using flow cytometry from dissociated whole tumor cell suspensions or cultured cells. T cells were identified through extracellular staining with markers specific to mouse CD45, CD3ε/TCRβ, CD4, CD8a, PD-1, and TIM-3. Myeloid-derived suppressor cells (MDSC) were identified through extracellular staining with markers specific to mouse CD45, CD11b, and Gr-1. Macrophage cells were identified through extracellular staining with markers specific to mouse CD45, CD11b and F4-80. Neutrophils cells were identified through extracellular staining with markers specific to mouse CD45, CD11b and Ly6G. Monocytes were identified through extracellular staining with markers specific to mouse CD45, CD11b and Ly6C.

### GSH/GSSG ratio, MDA measurement

The GSH/GSSG ratios and MDA levels were determined using a GSH/GSSG Ratio Assay Kit and an MDA kit (Beyotime, S0053; S0131S) in strict accordance with the manufacturer’s instructions.

### Animal studies

C57BL/6 (6–7 weeks old) and BALB/c male nude mice (4–5 weeks old) were obtained from Charles River Co., Ltd. The mice were maintained in a specific pathogen-free facility at the Laboratory Animal Research Center under controlled conditions (22.2 °C, 40–70% humidity, and a 12-hour light/dark cycle). All animal care procedures and experiments adhered to the *Guide for the Care and Use of Laboratory Animals* and were approved by the Institutional Animal Care and Use Committee of the Experimental Animal Center at Hebei Medical University, China. LLC-BIN1^KO^ and LLC-BIN1^WT^ cells (5 × 10⁵ cells in 100 µL PBS) were subcutaneously injected into the left flank of the nude mice. Tumor dimensions (short and long diameters) were measured with calipers every three days. Thirteen days post-injection, the C57BL/6 tumor-bearing mice were euthanized, while sixteen days post-injection, the Balb/c nude tumor-bearing mice were euthanized. Tumor volume was calculated using the formula: [volume = (width × width × length) / 2].

When the tumor volumes reached 100–150 mm³, the mice were randomly divided into two groups. One group was administered 20 mg/kg of SB02024 via oral gavage, while the other group received an equivalent volume of a 0.5% carboxymethyl cellulose solution containing 1% polysorbate 80, also via oral gavage. Mice that either failed to develop tumors or exhibited tumor volumes exceeding the predefined threshold of ≥ 2000 mm³, as stipulated by the in vivo experimental protocols approved by the Laboratory Animal Welfare and Ethics Committee, were excluded from the study.

### Co-immunoprecipitation (Co-IP)

Cell pellets were collected via centrifugation and resuspended in a lysis buffer supplemented with a protease inhibitor cocktail. The cells were lysed on ice for 30 min, followed by centrifugation at 15,000 rpm for 15 min. The resulting supernatants were collected for further analysis. Immunoprecipitation was performed using 5 µg of antibodies and 500 µg of proteins. BeyoMag™ Protein A + G Magnetic Beads (Beyotime Biotechnology) were employed to capture the precipitated proteins overnight at 4 °C. After incubation, the magnetic beads were washed three times with Tris-buffered saline to ensure thorough purification. Elution was carried out by boiling the magnetic beads in Laemmli sample buffer. The Co-IP products, along with their respective input samples, were then analyzed using western blotting. The antibodies utilized in this experiment included BIN1 (ab27796, Abcam), G3BP1 (ab56574, Abcam), STAT1 (9172, CST), and anti-Rabbit IgG (2729, CST).

### Molecular Docking analysis

The proteins analyzed in this study include Bin1 (ID: O08539), G3BP1 (ID: A0A6P5QR56), and STAT1 (ID: P42225). Their structures were predicted using AlphaFold3, and the models with the highest confidence scores were selected for subsequent docking analyses. The PDB files were preprocessed using PYMOL software to remove solvent molecules and extraneous ions that could interfere with the docking process. Protein-protein docking was performed using the HDOCK program, which employs a hybrid docking strategy combining template-based modeling and free docking to predict protein complex structures effectively. Global docking simulations were conducted to explore potential binding sites across the entire protein surfaces, generating multiple candidate complex models. Among these, the model with the most negative docking score was chosen as the final candidate, as lower docking scores generally correlate with higher likelihood of representing the true binding state. Finally, the selected complex model was subjected to interaction analysis using PyMOL (Version 3.0.3) to elucidate the molecular mechanisms underlying protein binding, including hydrogen bonds, hydrophobic interactions, and salt bridges.

### Isolation and stimulation of CD8^+^T cells

T cells were isolated from the spleens of C57BL/6 mice using a magnetic bead separation kit, following the manufacturer’s instructions. After T cells were isolated, they were cultured in RPMI1640 complete medium containing 10 ng ml^− 1^ IL-2. CD8^+^T cells were preincubated with LLC-BIN1^KO^ and LLC-BIN1^WT^ cells (at a ratio of 1:4) for 24 h. Following this, the cells were purified and activated using anti-CD3/CD28 beads (bead-to-cell ratio of 2:1, Thermo Fisher Scientific) [21]. On day 6 post-activation, the functional capacity of T cells was evaluated by quantifying cytotoxic cytokines and cytolytic activity via flow cytometry.

### CD8^+^T cell cytotoxicity assay

LLC cells were respectively co-cultured with CD8^+^T cells (It has been co-incubated with LLC-BIN1^KO^ and LLC-BIN1^WT^ cells for 6 days) for 48 h, and the apoptosis of LLC cells was detected by Annexin V-FITC apoptosis detection kit (#C1062S).

### Cytokine release assay

To analyze the cytokines such as IFN-γ, TNF-α and GZMB within CD8^+^T cells after co-culture with LLC-BIN1^KO^ and LLC-BIN1^WT^, cells were first harvested and washed twice with PBS. Then, the cells were fixed, permeabilized and stained using the intracellular fixation and permeabilization buffer kit (BD, HY-000544) according to the manufacturer’s protocol. Before flow cytometry analysis, the stained cells were washed twice again with PBS.

### HE staining

Initially, mouse tumor tissues were fixed using 4% paraformaldehyde. Following fixation, they underwent dehydration with alcohol, were embedded in paraffin, and sliced into 5 μm thick sections. The sections were then dewaxed, rehydrated, and rinsed with double-distilled water. Subsequently, the tissues were stained with hematoxylin for 8 min and rinsed under water. Afterward, they were immersed in 1% acidic alcohol for 5 min and again rinsed under running water. The sections were next stained with eosin for 3 min, dehydrated, and cleared using xylene. Finally, the prepared tissue sections were mounted with a mounting medium and observed under an optical microscope for analysis.

### Measurement of cytokines

Xenograft tumors were homogenized using a homogenizer, and cytokine levels in the tumor extracts were quantified with the CXCL10 Mouse Uncoated ELISA Kit and the CCL5 Mouse Uncoated ELISA Kit (Thermo Fisher Scientific). The data were standardized relative to the protein concentrations in the tumor extracts.

### RNA transcriptome sequencing

First, 1 µg of total RNA was dissolved in nuclease-free H₂O to a final volume of 50 µL in nuclease-free PCR tubes and stored at -20 °C. mRNA capture beads were utilized to isolate mRNA from the sample for fragmentation. Double-stranded cDNA was synthesized and subsequently purified using magnetic beads. End repair and end dA-tailing processes were carried out, followed by the addition of RNA adapters to the sample for the connection reaction. VAHTS DNA clean beads were then introduced to the connected product to bind cDNA to the magnetic beads, facilitating product purification and fragment size selection. The PCR Primer Mix and Amplification Mix 1 were employed to amplify the library, and the resulting PCR products were purified using magnetic beads. Finally, the library was quantified for chip preparation, after which the chip was loaded into the analyzer for verification.

### Untargeted metabolomics by high-performance liquid chromatography-mass spectrometry

Metabolic extracts were obtained from LLC-BIN1^KO^ and LLC-BIN1^WT^ cells using methanol-assisted protein precipitation. These extracts were subsequently analyzed by liquid chromatography-mass spectrometry (LC-MS) on a UHPLC System (1290, Agilent Technologies, Santa Clara, California, USA) equipped with a UPLC BEH Amide column for separation. The mobile phase comprised 25 mmol/L ammonium acetate and 25 mmol/L ammonium hydroxide in water, combined with acetonitrile. A triple time-of-flight (TOF) mass spectrometer (AB Sciex) was employed for its capability to acquire MS/MS spectra through information-dependent acquisition during LC/MS experiments. In this mode, the acquisition software (Analyst TF 1.7, AB Sciex) continuously evaluated full-scan survey MS data in real time, triggering the acquisition of MS/MS spectra based on preselected criteria. During each cycle, the 12 most intense precursor ions with an intensity greater than 100 were selected for MS/MS analysis at a collision energy of 30 eV.

The MS raw data files were converted to the mzXML format using ProteoWizard and subsequently processed with the R package XCMS (v3.2). This workflow included peak deconvolution, alignment, and integration. The parameters for Minfrac and cut-off were set to 0.5 and 0.3, respectively. An in-house MS2 database was utilized for metabolite identification.

### Flow cytometry mass spectrometry analysis

To identify the key effector cells driving the protumoral immunity resulting from BIN1 knockout, CyTOF experiments were conducted. For CyTOF sample preparation, 3 × 10^6 cells were transferred to a flow cytometry tube and washed twice with calcium- and magnesium-free PBS by centrifuging at 500 g for 5 min at room temperature. To differentiate between dead and live cells, 1 mL of a 0.5 µM platinum dichloride solution was added, mixed thoroughly, and incubated at room temperature for 2 min.

### Immunofluorescence assay

Tumors were post-fixed in 4% paraformaldehyde at 4 °C for 24–48 h and then dehydrated in a 30% sucrose solution for 48 h. Tumor sections were prepared along the coronal plane at a thickness of 25 μm using a freezing microtome (Leica Microsystems, Germany). The sections were subsequently washed in PBS (3 × 5 min) and blocked in 5% bovine serum albumin (BSA) with 0.25% Triton X-100 in PBS for 30 min at room temperature. Following this, they were incubated overnight at 4 °C with primary antibodies diluted in 2% BSA-PBS. The primary antibody used was rabbit anti-CD8 (Abcam, US). After thorough PBS washing, the sections were incubated with appropriate fluorescently conjugated secondary antibodies for 2 h at room temperature on a low-speed shaker. Finally, the sections were mounted onto slides. Fluorescence images were captured using a confocal microscope (Zeiss, Germany) and processed with ZEN software (Zeiss). Cell counts were performed manually using the confocal images.

### Statistical analysis

Data were presented as mean ± SD. All statistical analyses were conducted using SPSS version 23 or GraphPad Prism version 10.0 (GraphPad Software, San Diego, California, USA). Differences in cell viability were evaluated through two-way analysis of variance. To investigate metabolic alterations among groups, a supervised partial least squares discrimination analysis (PLS-DA) model was employed. Pathway enrichment analysis was carried out using the KEGG database. Qualitative and quantitative differences between subgroups were assessed using the χ² test or Fisher’s exact test for categorical variables. For comparisons between two groups, Student’s t-test or the Mann-Whitney U test was applied, as appropriate. Statistical significance was defined as *P* < 0.05.

## Results

### BIN1 downregulation predicts advanced progression and poor prognosis in NSCLC

**Fig. 1:**
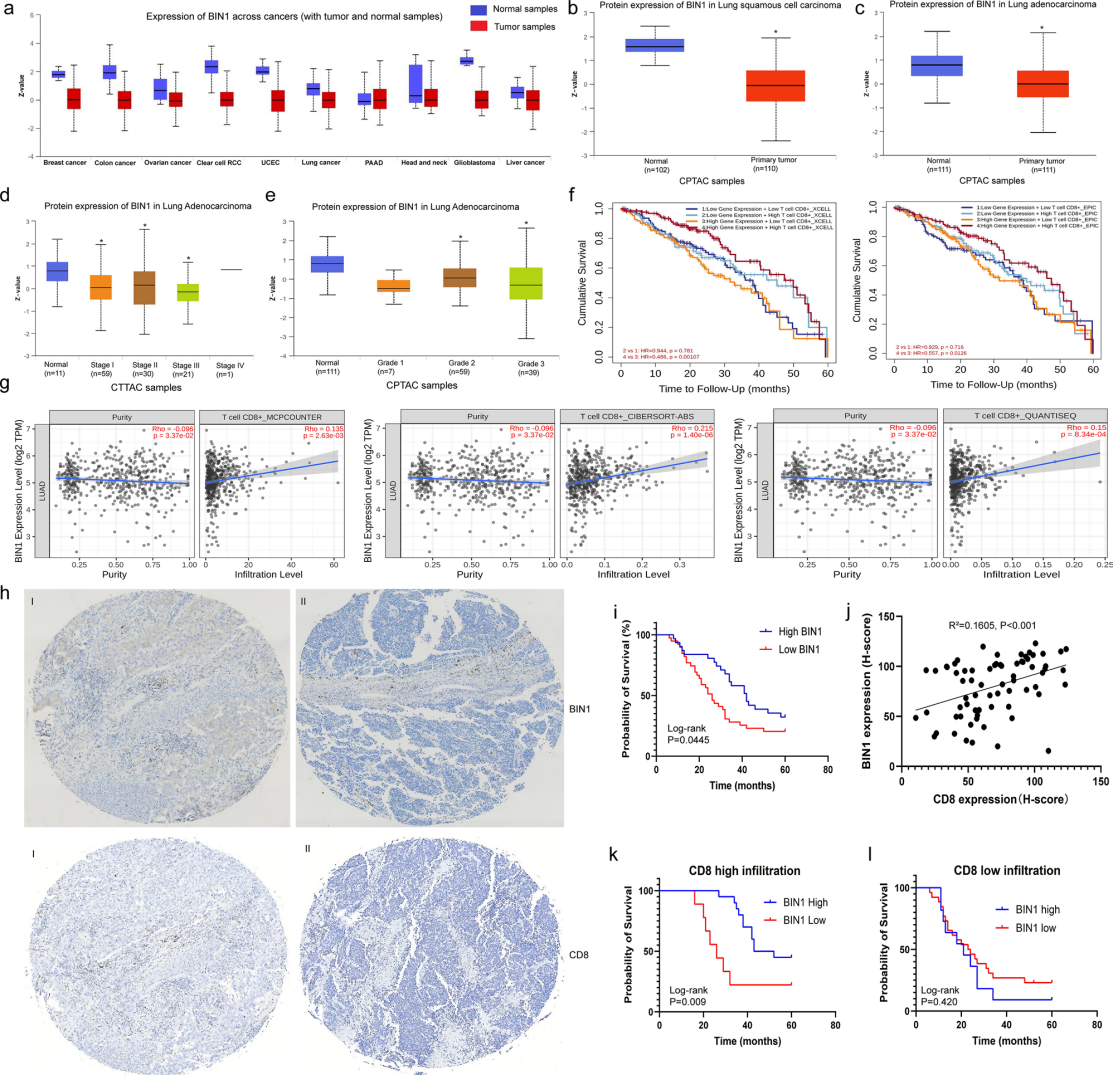
BIN1 Attenuation Predicts Advanced Progression and Poor Prognosis in NSCLC **a**: Protein expression levels of BIN1 across various cancers, as analyzed using the CPTAC dataset. **b**: Protein expression levels of BIN1 in LUSC. **c**: Protein expression levels of BIN1 in LUAD. **d-e**: The relationship between BIN1 expression levels and both clinical stages and histological differentiation degrees. **f**: The correlation between BIN1 expression and overall survival (OS) in NSCLC patients. **g**: The combined impact of BIN1 and CD8 co-expression on the prognosis of NSCLC patients. **h**: Tissue microarray analysis of BIN1 and CD8 expression levels in NSCLC samples (I: BIN1 high expression. II: BIN1 low expression. Scale bar = 10 μm). **i**: Kaplan-Meier analysis illustrating the association between BIN1 expression and the prognosis of NSCLC patients. **j**: Spearman correlation analysis was conducted to explore the relationship between BIN1 and CD8^+^ T cell infiltration in NSCLC. **k-l**: Kaplan-Meier survival analysis was used to observe the correlation between the expression level of BIN1 and the prognosis of NSCLC patients under different CD8^+^ T cell infiltration states

We analyzed the protein expression levels of BIN1 across various cancers using the CPTAC dataset. BIN1 was found to be downregulated in breast cancer, colon cancer, ovarian cancer, clear cell renal cell carcinoma (RCC), uterine corpus endometrial carcinoma (UCEC), lung cancer, glioblastoma, and liver cancer, suggesting its potential role as a key regulator in cancer progression (Fig. 1a). Additionally, further analysis of the CPTAC database revealed significantly reduced BIN1 expression levels in both lung adenocarcinoma (LUAD) and lung squamous cell carcinoma (LUSC) (Fig. 1b and c). Moreover, lower BIN1 expression was strongly associated with advanced clinical stages and higher histologic grades, as evidenced in the CPTAC database based on TCGA data (Fig. 1d and e) (Supplementary Fig. 1a-c). These findings indicate that reduced BIN1 expression is closely linked to advanced progression in NSCLC.

To elucidate the clinical significance of BIN1 in lung cancer, we analyzed the correlation between BIN1 expression levels and the prognosis of lung cancer patients using the StarBase2.0 and TIMER2.0 databases, both derived from TCGA data. The results indicated that BIN1 expression levels were not significantly associated with the overall survival (OS) of LUAD patients (Fig. 1f, Supplementary Fig. 1d). However, a noteworthy finding from the TIMER2.0 database revealed that NSCLC patients exhibiting both high BIN1 and high CD8 expression demonstrated significantly improved OS compared to those with high BIN1 but low CD8 expression (Fig. 1f). Further analysis was conducted to investigate the relationship between BIN1 expression and CD8^+^ T cell infiltration using Spearman correlations within the TIMER2.0 database. Additionally, results from CIBERSORT, MCP-counter, and QUANTISEQ analyses consistently showed that BIN1 was positively correlated with CD8^+^ T cell infiltration levels (Fig. 1g). Taken together, these findings suggest that the regulatory role of BIN1 in NSCLC is intricately linked to CD8^+^ T cell infiltration.

A tissue microarray consistently revealed a significant decrease in BIN1 expression within NSCLC tissues (Fig. 1h, Supplementary 1e). Furthermore, low BIN1 expression was strongly associated with lymph node metastasis and more advanced clinical stages (Table 1). Kaplan-Meier survival analysis further demonstrated that NSCLC patients with diminished BIN1 expression experienced shorter overall survival (OS) times (Fig. 1i). To further investigate the association between BIN1 expression status and CD8^+^ T cell infiltration, we evaluated CD8 expression in NSCLC tissues using immunohistochemistry. Spearman correlation analysis revealed a significant positive correlation between BIN1 expression and CD8^+^ T cell infiltration (R^2^ = 0.1605, *P* < 0.001) (Fig. 1j). Additionally, we investigated the association between BIN1 expression and patient prognosis across different stages and varying CD8^+^ T cell infiltration statuses. Kaplan-Meier analysis showed that in stage I/II lung cancer, high BIN1 expression correlated with improved survival compared to low expression (Supplementary Fig. 1g). In stage III, BIN1 expression was not significantly associated with prognosis (Supplementary Fig. 1h). Furthermore, in cases with CD8^+^ T cell infiltration, high BIN1 expression was linked to better prognosis. Conversely, in low CD8^+^ T cell infiltration cases, BIN1 expression did not significantly affect prognosis (Fig. 1k and l). These results suggest that low BIN1 expression is significantly associated with poor prognosis and reduced CD8^+^ T cell infiltration in NSCLC patients.

**Table 1:** The relationship between BIN1 expression and clinicopathologic features in lung cancer

Features	*n*	BIN1 expression status [*n*(%)]		χ2	*P*-Value
		high (*n* = 31)	low (*n* = 39)		
Age				1.100	0.294
<50 years	32	12(37.5)	20(62.5)		
≥50 years	38	19(50.0)	19(50.0)		
Sex				0.320	0.572
Male	41	17(41.46)	24(58.54)		
Female	29	14(48.28)	15(51.72)		
Tumor size				3.261	0.071
≤2 cm	30	17(56.67)	13(43.33)		
>2 cm	40	14(35.0)	26(65.0)		
Lymph node metastasis				7.566	0.006^**^
Negative	28	18(64.29)	10(35.71)		
Positive	42	13(30.95)	29(69.05)		
Differentiation grade				3.412	0.064
I	32	18(56.25)	14(43.75)		
II ~ III	38	13(34.21)	25(65.79)		
TNM stage				5.5054	0.0190^*^
I ~ II	46	25(54.35)	21(45.65)		
III	24	6(25.00)	18(75.00)		

Note: ^*^P < 0.05, ^**^P < 0.01

### Knockout of BIN1 inhibits the expression of immune-related genes and the activation of immune-related pathways

We utilized CRISPR-Cas9 technology in the murine NSCLC line LLC to generate clonal variants with BIN1 loss-of-function mutations (Supplementary Fig. 2a-d), referred to hereafter as LLC-BIN1^KO^. EdU incorporation, wound healing, Transwell migration, and invasion assays demonstrated that BIN1 knockout markedly enhanced the proliferation, migration, and invasion of LLC cells (Supplementary Fig. 2e-h). Moreover, BIN1 knockout significantly reduced the apoptosis rate of LLC cells, as indicated by additional analysis (Supplementary Fig. 2i).

**Fig. 2:**
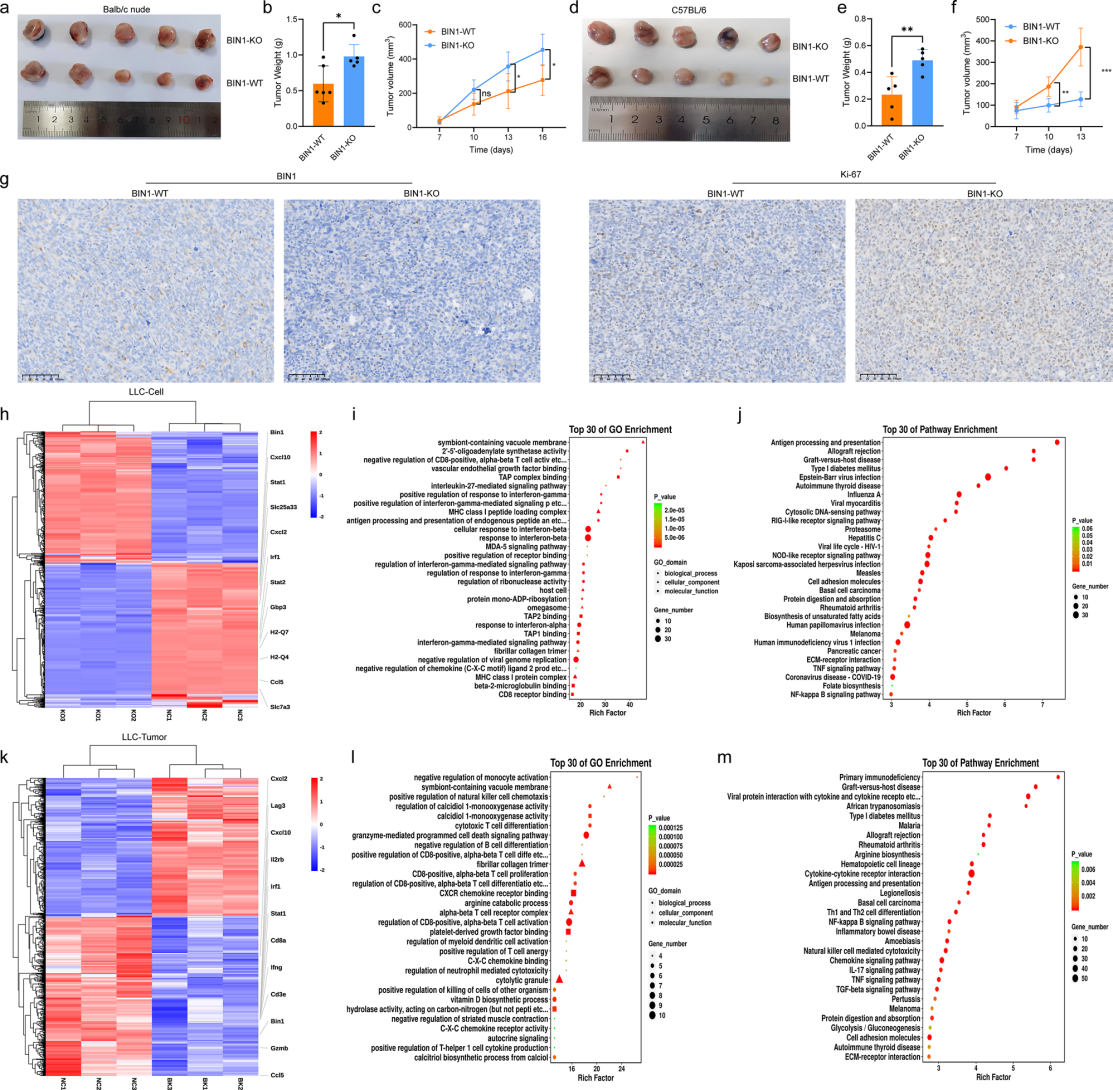
BIN1 Knockout Suppresses the Expression of Immune-Related Genes and Inhibits the Activation of Immune-Related Pathways **a-c**: The impact of BIN1 knockout on tumorigenicity in nude mice. **d-f**: The impact of BIN1 knockout on tumorigenicity in C57BL/6 mice. **g**: Immunohistochemical analysis of Ki67 expression in tumor tissues from C57BL/6 mice across different groups. **h**: Heatmap illustrating differentially expressed genes between BIN1 knockout and control LLC cells. **i**: GO enrichment analysis of differentially expressed genes between BIN1 knockout and control LLC cells. **j**: KEGG pathway analysis of differentially expressed genes between BIN1 knockout and control LLC cells. **k**: Heatmap depicting differentially expressed genes between BIN1 knockout and control tumors. **l**: GO enrichment analysis of differentially expressed genes between BIN1 knockout and control tumors. **m**: KEGG pathway analysis of differentially expressed genes between BIN1 knockout and control tumors

Furthermore, to investigate the biological function of BIN1 in vivo, we injected LLC-BIN1^WT^ and LLC-BIN1^KO^ cells into nude mice (*n* = 5 mice/group) to establish subcutaneous mouse models. The growth rate and average tumor weights derived from LLC-BIN1^KO^ group cells were significantly faster and heavier than those of the control (Fig. 2a-c). Previous studies have demonstrated that BIN1 is closely associated with CD8^+^ T cell infiltration. Therefore, we utilized immunocompetent C57 mice to investigate the in vivo regulatory role of BIN1 (*n* = 5 mice/group). The results revealed that the tumor growth rate and tumor weight in the BIN1 knockout group were significantly higher compared to those in the control group (Fig. 2d-f). Meanwhile, IHC staining for Ki-67, a proliferation marker, was significantly increased in orthotopic tumors of the LLC-BIN1^KO^ group (Fig. 2g). In summary, our findings demonstrate that BIN1 loss-of-function mutations enhance LLC cell proliferation, migration, and invasion, while also promoting tumor growth and reducing apoptosis, both in vitro and in vivo.

To elucidate the genome-wide expression patterns regulated by BIN1, we conducted RNA-seq analysis in paired LLC-BIN1^KO^ and LLC-BIN1^WT^ C57BL/6 allograft tumors in vivo and LLC-BIN1^KO^ and LLC-BIN1^WT^ cells in vitro. Kyoto Encyclopedia of Genes and Genomes (KEGG) and Gene Ontology (GO) analyses revealed that gene sets involved in immune response regulation, cytokine production, interferon-γ (IFN-γ) response, and antigen processing and presentation were significantly downregulated in both LLC-BIN1^KO^ tumors and LLC-BIN1^KO^ cells (Fig. 2h-m). The IFN-γ pathway plays a critical role in the tumor’s response to immunotherapy. In LLC-BIN1^KO^ tumors and LLC-BIN1^KO^ cells, the key transcription factor STAT1 in the IFN-γ pathway was markedly downregulated (Fig. 2h and k). Additionally, BIN1 inactivation led to decreased expression of IFN-γ and its induced chemokine genes CXCL10 and CCL5 both in vitro (Fig. 2h) and in vivo (Fig. 2k). The STAT1-IFNγ regulatory axis has been reported to enhance anti-tumor immunity by recruiting CXCR3^+^CD8^+^ T cells and CXCR3^+^ NK cells [22]. Collectively, these findings suggest that BIN1 knockout leads to decreased STAT1 expression, consequently suppressing the IFN-γ pathway, downregulating immune-related gene expression, and impairing the activation of immune-related pathways.

### Deletion of BIN1 inhibits CD8^+^ T cell infiltration and impairs their cytotoxic function in the NSCLC TME

**Fig. 3:**
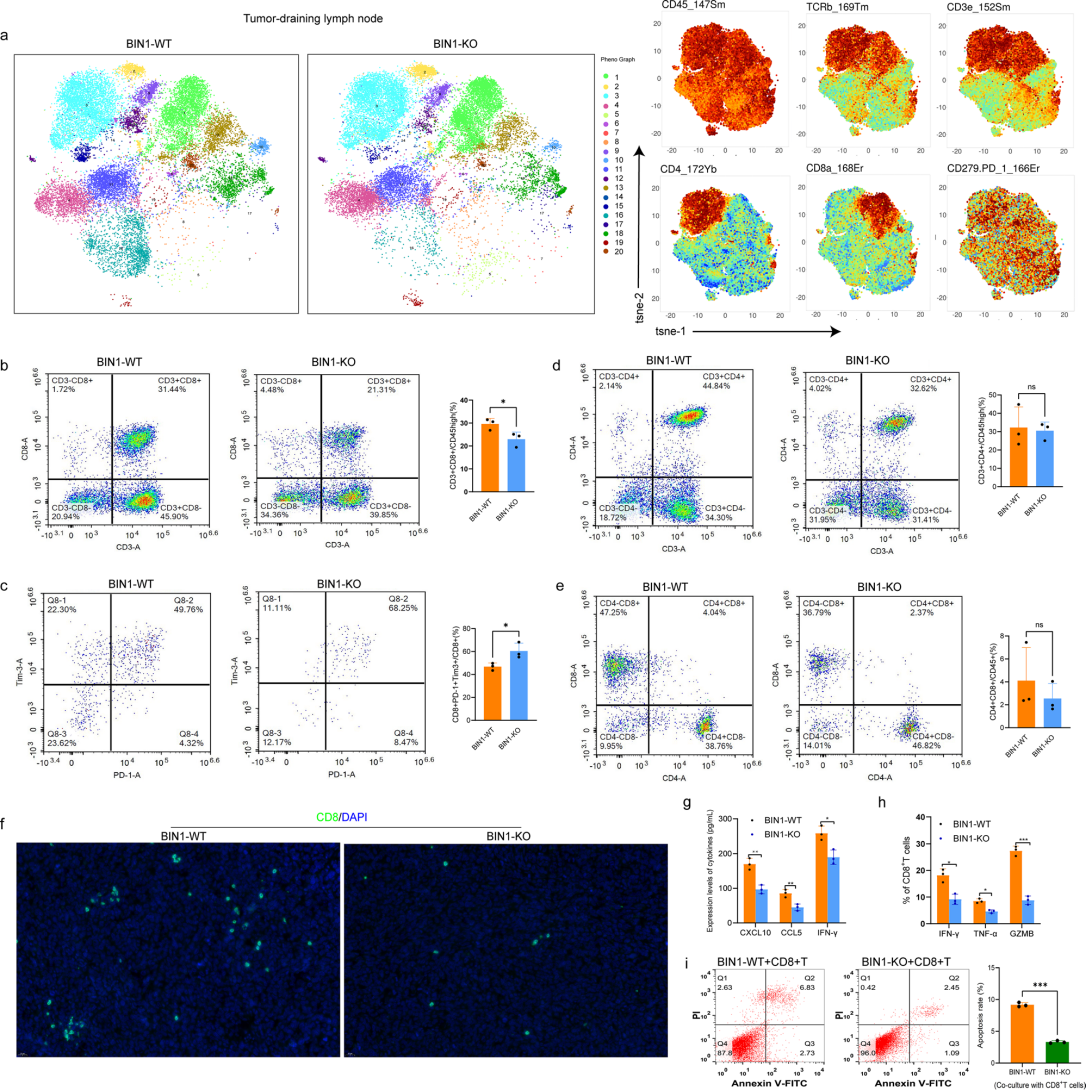
Deletion of BIN1 Inhibits CD8^+^ T Cell Infiltration and Impairs Their Cytotoxic Function in the NSCLC TME **a**: Flow cytometry mass spectrometry analysis was conducted on tumor-draining lymph nodes from BIN1-KO and BIN1-WT mice (1: CD8^+^ T. 2–3: CD4^+^ T. 4, 5, 6, 11, 13, 16, 17, 18, 19, 20: B cell. 7: MDSC. 8: Neutrophils. 9, 12, 14, 15: T cell. 10: DC). **b**: The proportion of CD8^+^ T cells in tumor tissues was analyzed across each group of mice using flow cytometry. **c**: Flow cytometry detected the proportion of exhausted CD8^+^ T cells in the tumor tissues of each group of mice. **d**: The proportion of CD4^+^ T cells in tumor tissues from each group of mice was assessed via flow cytometry. **e**: Flow cytometry identified the proportion of CD4^+^ CD8^+^ T cells in the tumor tissues of each group of mice. **f**: Tissue immunofluorescence was used to evaluate the infiltration level of CD8^+^ T cells in the tumor tissues from each group of mice. **g**: The effect of BIN1 knockout on CXCL10, CCL5 and IFN-γ expression in mouse tumor tissues were determined. **h**: Flow cytometry was employed to assess the alterations in cytotoxic factors within CD8^+^ T cells following co-culture with LLC-BIN1^WT^ and LLC-BIN1^KO^ cells. **i**: Flow cytometry was employed to assess the cytotoxic activity of CD8^+^ T cells co-cultured with LLC-BIN1^WT^ and LLC-BIN1^KO^ cells against LLC cells

To further evaluate whether host BIN1 influenced the TME, we analyzed tumor-infiltrating immune cells in detail. Mass cytometry revealed a significant increase in CD8^+^ T cells within the draining lymph nodes of LLC-BIN1^KO^ mice compared to LLC-BIN1^WT^ mice (Fig. 3a; Supplementary 3a, b). Subsequently, multi-color flow cytometry was performed to assess immune cell infiltration in tumor tissues across each group of mice. In the TME of LLC-BIN1^KO^ mice, we observed a substantial decrease in CD8^+^ T cells and a concurrent increase in CD8^+^TIM3^+^PD-1^+^ T cells, relative to LLC-BIN1^WT^ mice (Fig. 3b, c). Other immune cell populations—such as CD4^+^ T cells, CD8^+^ T cells, M-MDSCs, macrophages, monocytes, neutrophils, and NK cells—showed minimal differences in the spleen, draining lymph nodes, and tumors (Fig. 3d, e; Supplementary 3a-g). Furthermore, immunohistochemical staining for CD8 on LLC tumor tissues from LLC-BIN1^KO^ mice verified a reduced presence of CD8^+^ T cells (Fig. 3f).

To further explore the effect of BIN1 on the TME in NSCLC, RNA sequencing analysis was conducted on tumor tissues from both BIN1-KO and BIN1-WT mice. The results demonstrated a significant reduction in the expression levels of CD8 surface markers CD8a and CD28 in the BIN1-KO group. Additionally, the expression of GZMB, a cytotoxic molecule crucial for CD8^+^ T cell functionality, was markedly reduced (Fig. 2k). These findings highlight the suppressed antitumor activity of CD8^+^ T cells within the TME of LLC-BIN1^KO^ mice.

RNA-seq analysis of LLC cells with BIN1 knockout compared to the control group revealed a significant reduction in the expression levels of STAT1, IFN-γ, CXCL10, and CCL5, which are key factors involved in CD8^+^ T cell chemotaxis (Fig. 2h, k). To validate these findings, we measured the expression levels of IFN-γ, CXCL10, and CCL5 in LLC-BIN1^KO^ and LLC-BIN1^WT^ mice using ELISA. The results confirmed that the BIN1 knockout group exhibited markedly lower levels of IFN-γ, CXCL10, and CCL5 compared to the control group (Fig. 3g).

To investigate the effect of BIN1 knockout in tumor cells on CD8^+^ T cell function, LLC-BIN1^WT^ and LLC-BIN1^KO^ cells were co-cultured with CD8^+^ T cells. After 6 days, flow cytometry was employed to evaluate the expression levels of cytotoxic factors (IFN-γ, TNF-α, and GZMB) in CD8^+^ T cells from each co-culture system. The results demonstrated that the expression levels of IFN-γ, TNF-α, and GZMB were significantly reduced in CD8^+^ T cells co-cultured with LLC-BIN1^KO^ compared to those co-cultured with LLC-BIN1^WT^ (Fig. 3h). Furthermore, CD8^+^ T cells isolated from each co-culture system were re-co-cultured with LLC cells, and the apoptosis rate of LLC cells was assessed by flow cytometry. The findings revealed that CD8^+^ T cells co-cultured with LLC-BIN1^KO^ exhibited decreased cytotoxic activity against LLC cells compared to those co-cultured with LLC-BIN1^WT^ (Fig. 3i). These observations led to the hypothesis that BIN1 deficiency facilitates tumor progression by modifying the TME, thereby impairing the antitumor response.

### BIN1 modulates STAT1 stabilization by competitively binding to G3BP1

**Fig. 4:**
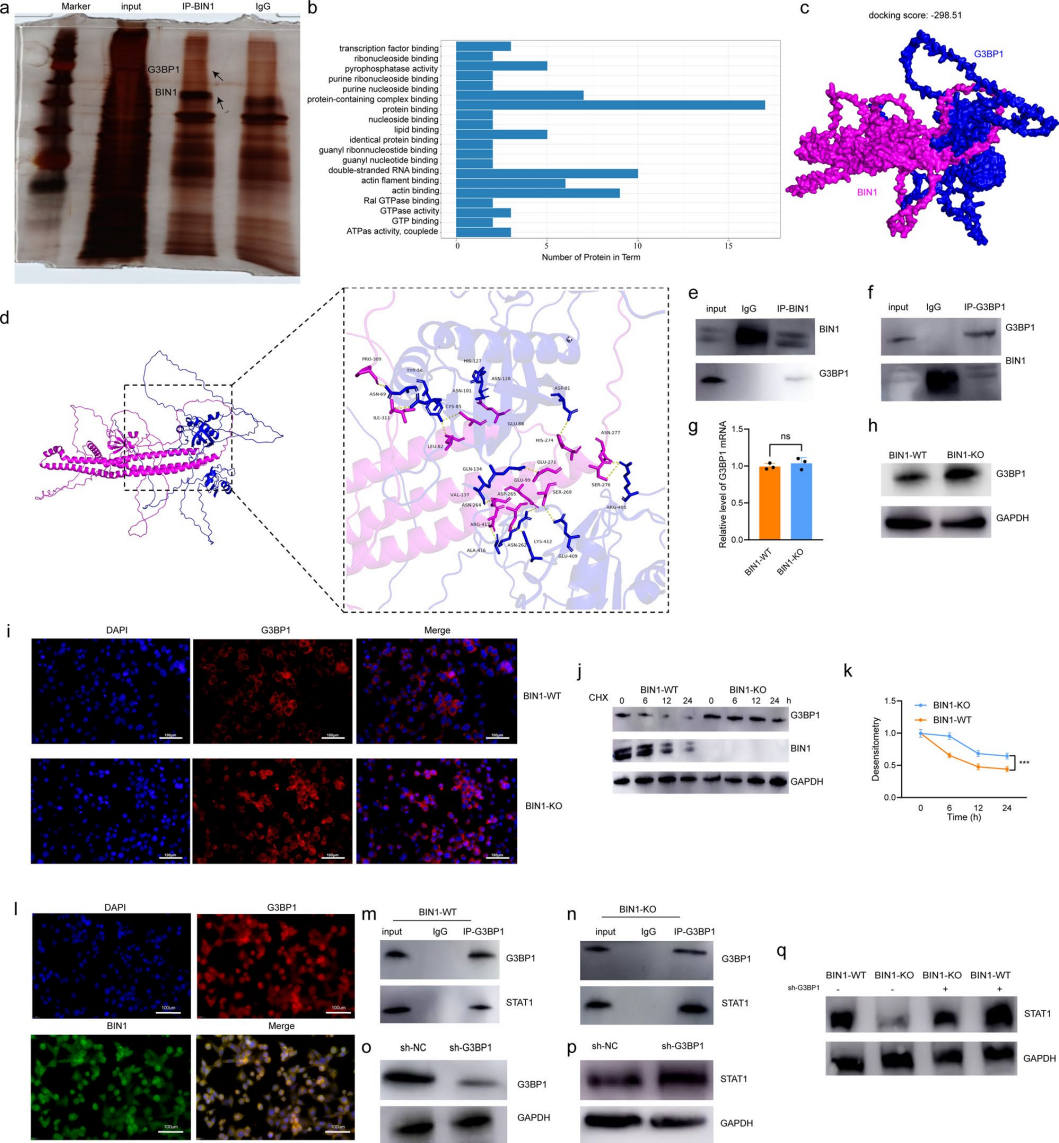
BIN1 Modulates STAT1 Stabilization by Competitively Binding to G3BP1 **a**: Immunoprecipitation-mass spectrometry analysis identifying the interacting partners of BIN1. **b**: GO analysis of differentially expressed proteins in the IgG and IP groups. **c**: Molecular docking experiments assessing the binding potential and identifying the interaction sites between BIN1 and G3BP1. **d**: Identification of the binding sites between BIN1 and G3BP1. **e-f**: Co-IP confirming the interaction between BIN1 and G3BP1. **g**: Analysis of the regulatory effect of BIN1 knockout on the transcriptional level of G3BP1. **h**: Analysis of the regulatory effect of BIN1 knockout on the protein level of G3BP1. **i**: Immunofluorescence assay detecting the effects of BIN1 knockout on G3BP1. **j-k**: Investigation of G3BP1 protein stability in the presence of BIN1 following CHX intervention. **l**: Immunofluorescence assay examining the co-localization of BIN1 and G3BP1. **m**: Co-IP validation of the interaction between STAT1 and G3BP1 in BIN1 wild-type (WT) cells. **n**: Co-IP validation of the interaction between STAT1 and G3BP1 in BIN1 knockout (KO) cells. **o-p**: Western blot analysis determining the effect of G3BP1 knockdown on STAT1 expression. **q**: BIN1 influences STAT1 expression through G3BP1

To investigate the role of BIN1 in regulating CD8^+^ T cell infiltration, we conducted immunoprecipitation-mass spectrometry to identify its interacting partners (Fig. 4a). GO analysis of the differentially expressed proteins between the IgG group and the IP group revealed that the potential binding partners of BIN1 were associated with GTPase binding, GTPase activity, and GTP binding (Fig. 4b). Among these, we focused on Ras-GTPase-activating protein-binding protein 1 (G3BP1). To determine whether BIN1 binds to G3BP1, we first performed molecular docking experiments to assess their binding potential and identify the binding sites between BIN1 and G3BP1 (Fig. 4c). The docking analysis produced a score of -298.51, suggesting a strong interaction between BIN1 and G3BP1. Further investigation revealed that their interaction is primarily facilitated by hydrogen bonds and salt bridges (Fig. 4d). G3BP1, a stress granule (SG) assembly factor, has been implicated in SG formation and cancer progression [23], making it a key protein of interest. For further validation, Co-IP assays were performed and confirmed that endogenous BIN1 interacts with G3BP1 in LLC cells (Fig. 4e and f). Additionally, database analyses showed that G3BP1 expression is significantly upregulated in NSCLC tissues and correlated with advanced clinical stages. However, no significant association was observed between G3BP1 expression and the prognosis of NSCLC patients (Supplementary Fig. 4b, d and f). These findings highlight the importance of the BIN1-G3BP1 interaction and its potential implications in cancer progression.

We next investigated whether BIN1 influences the expression of G3BP1. Western blotting and immunofluorescence (IF) demonstrated that G3BP1 protein expression was significantly upregulated upon BIN1 knockout (Fig. 4h and i). However, no regulatory effect was observed at the transcriptional level (Fig. 4g). Moreover, BIN1 knockout was found to preserve G3BP1 stability in the presence of the protein synthesis inhibitor cycloheximide (CHX) (Fig. 4j and k). This interaction was further validated by the colocalization of BIN1 and G3BP1 in the cytoplasm, as shown through IF staining (Fig. 4l). These findings indicate that BIN1 knockout markedly enhances the stability of the G3BP1 protein.

To further investigate the potential mechanism through which BIN1/G3BP1 influences the TME, we systematically analyzed the potential binding proteins of G3BP1 using databases BioGRID and IntAct, and further investigated the interaction between G3BP1 and STAT1 through molecular docking experiments, the docking score was − 246.23 (Supplementary Fig. 4a). To confirm this interaction, we conducted Co-IP experiments, which demonstrated that G3BP1 indeed binds to STAT1 (Fig. 4m). Interestingly, we observed that the binding affinity between G3BP1 and STAT1 was significantly enhanced in BIN1-knockout LLC cells (Fig. 4n). Western blot analysis demonstrated that G3BP1 knockdown significantly upregulated STAT1 expression (Fig. 4o and p). Additionally, Western blot results demonstrated that BIN1 knockout inhibited STAT1 expression, an effect that was antagonized by G3BP1 knockdown (Fig. 4q). The above results indicate that BIN1 knockout enhances the interaction between G3BP1 and STAT1 by stabilizing G3BP1.

To verify the clinical significance of STAT1 in NSCLC, we analyzed publicly available databases and found that STAT1 expression was significantly downregulated in NSCLC. However, the expression level of STAT1 was not significantly correlated with either the clinical stage or prognosis in NSCLC patients (Supplementary Fig. 4c, e and g). Collectively, these findings suggest that BIN1 regulates STAT1 expression by competing with G3BP1 for binding interactions.

### BIN1 knockout inhibits CD8^+^T cell infiltration by inactivating the STAT1 signaling pathway

**Fig. 5:**
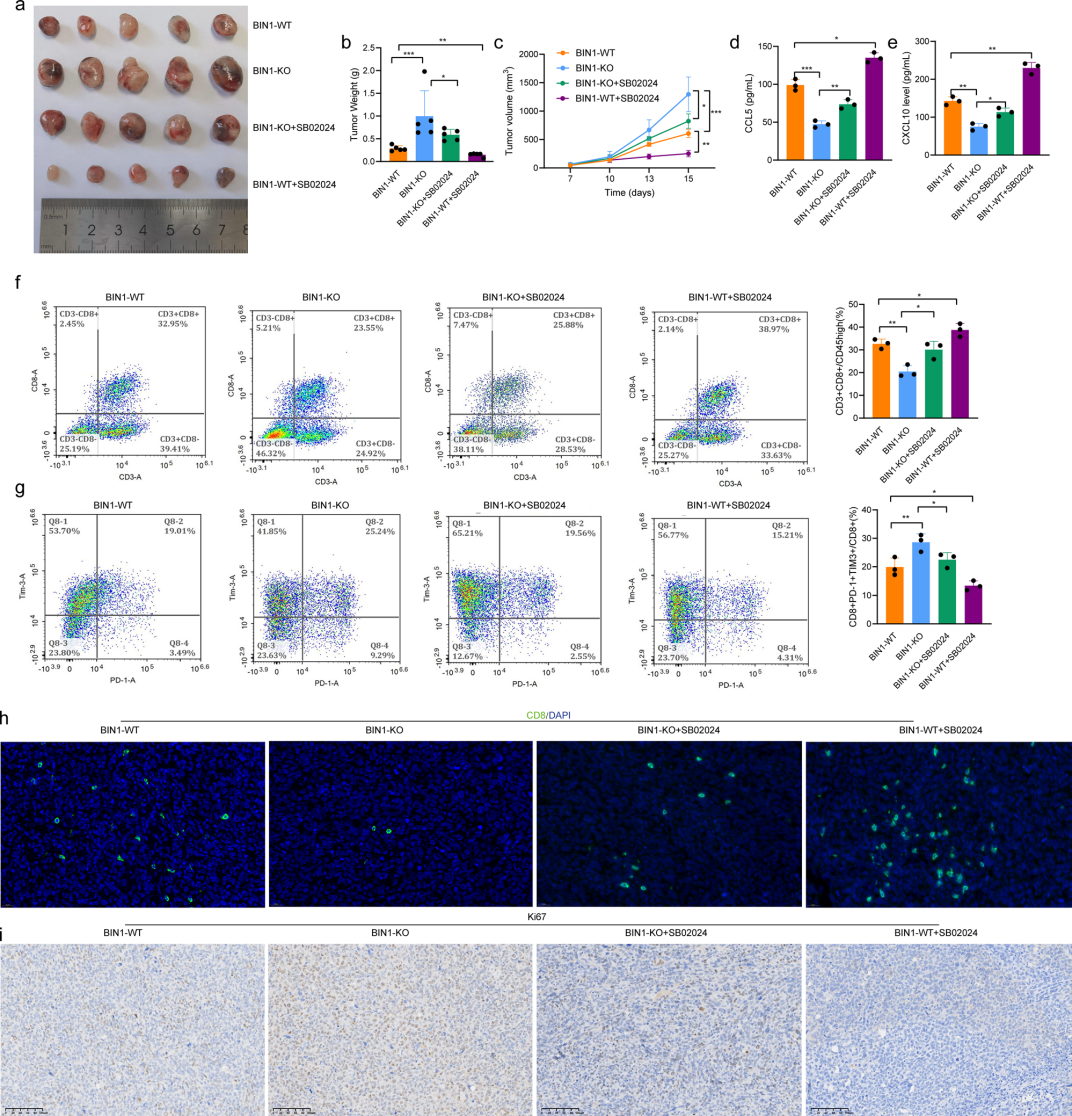
BIN1 Knockout Inhibits CD8^+^ T Cell Infiltration by Inactivating the STAT1 Signaling Pathway **a-c**: The impact of the STAT1 agonist, SB02024, on the tumorigenic potential of BIN1-KO cells. **d**: The effect of the STAT1 agonist, SB02024, on CXCL10 expression in tumors derived from BIN1-KO cells. **e**: The influence of the STAT1 agonist, SB02024, on CCL5 expression in tumors derived from BIN1-KO cells. **f**: The role of the STAT1 agonist, SB02024, in modulating CD8^+^ T cell infiltration within tumors derived from BIN1-KO cells. **g**: The effect of the STAT1 agonist, SB02024, on the infiltration of exhausted CD8^+^ T cells in tumors derived from BIN1-KO cells. **h**: The effect of the STAT1 agonist, SB02024, on the infiltration of CD8^+^ T cells in tumors derived from BIN1-KO cells. **i**: The influence of the STAT1 agonist, SB02024, on the expression of Ki-67 in tumors derived from BIN1-KO cells

To investigate whether BIN1 influences CD8^+^ T cell infiltration through the STAT1 signaling pathway, subcutaneous tumor models were established using LLC cells in BIN1-KO and BIN1-WT groups. The STAT1 agonist SB02024 was administered intraperitoneally when tumor volumes reached 100 mm³. The results showed that SB02024 significantly inhibited the tumorigenic potential of LLC cells and counteracted the pro-tumorigenic effects of BIN1 knockout (Fig. 5a–c). ELISA analyses revealed that CXCL10 and CCL5 expression levels in tumor tissues were significantly lower in BIN1-KO mice compared to BIN1-WT mice (Fig. 5d, e). Furthermore, treatment of BIN1-KO mice with SB02024 markedly upregulated CXCL10 and CCL5 expression levels relative to untreated BIN1-KO mice (Fig. 5d, e). These findings indicate that BIN1 regulates the expression of the chemokines CXCL10 and CCL5 via the STAT1 signaling pathway. In addition, we evaluated the regulatory effects of the STAT1 agonist SB02024 on the immune microenvironment of NSCLC using multi-color flow cytometry. Compared to the BIN1-WT group, the proportion of CD8^+^ T cell infiltration in tumor tissues was increased in the BIN1-WT + SB02024 group, while the proportion of CD8^+^PD-1^+^TIM3^+^ T cell infiltration was reduced (Fig. 5f, g). Similarly, in the BIN1-KO group, SB02024 treatment elevated the proportion of CD8^+^ T cell infiltration and decreased the proportion of CD8^+^PD-1^+^TIM3^+^ T cell infiltration compared to untreated BIN1-KO mice (Fig. 5f, g). Tissue fluorescence staining further demonstrated that SB02024 increased the infiltration of CD8^+^ T cells in the TME and partially reversed the inhibition of CD8^+^ T cell infiltration caused by BIN1 knockout (Fig. 5h). IHC) analysis revealed that SB02024 significantly reduced Ki67-positive rates in tumor tissues. Specifically, the positive rate of Ki67 expression in the BIN1-WT + SB02024 group was markedly lower than in the BIN1-WT group. Similarly, the BIN1-KO + SB02024 group exhibited a significantly reduced Ki67-positive rate compared to the BIN1-KO group (Fig. 5i). In a word, these findings suggest that the STAT1 agonist SB02024 can reverse the immunosuppressive microenvironment induced by BIN1 knockout and enhance anti-tumor immune responses.

In addition, we examined whether the effects of BIN1 knockout on the malignant biological behavior of NSCLC were associated with STAT1. EdU assays demonstrated that, compared to the BIN1-WT group, cells in the BIN1-WT + SB02024 group exhibited a significantly reduced proliferative capacity. Similarly, cells in the BIN1-KO + SB02024 group displayed markedly decreased proliferation compared to the BIN1-KO group (Supplementary Fig. 5b). Scratch healing assays showed that, compared to the BIN1-WT group, cells in the BIN1-WT + SB02024 group exhibited notably diminished migratory ability. Consistently, cells in the BIN1-KO + SB02024 group also exhibited significantly reduced migration relative to the BIN1-KO group (Supplementary Fig. 5c). Furthermore, Transwell migration and invasion assays revealed that, compared to the BIN1-WT group, cells in the BIN1-WT + SB02024 group showed a pronounced reduction in migration and invasion capabilities. Similarly, cells in the BIN1-KO + SB02024 group exhibited significantly diminished migration and invasion when compared to the BIN1-KO group (Supplementary Fig. 5d). Collectively, these findings suggest that the STAT1 agonist SB02024 effectively suppresses the proliferation, migration, and invasion of NSCLC cells, partially counteracting the pro-tumorigenic effects induced by BIN1 knockout.

These results indicate that BIN1 influences the expression of CXCL10 and CCL5 in the tumor microenvironment by modulating STAT1, thereby suppressing CD8^+^ T cell infiltration and affecting tumor progression. However, the precise mechanisms through which BIN1 and STAT1 regulate the proliferation, migration, and invasion of NSCLC cells remain to be elucidated.

### BIN1 regulates ferroptosis in NSCLC cells via STAT1/GSH pathway

**Fig. 6:**
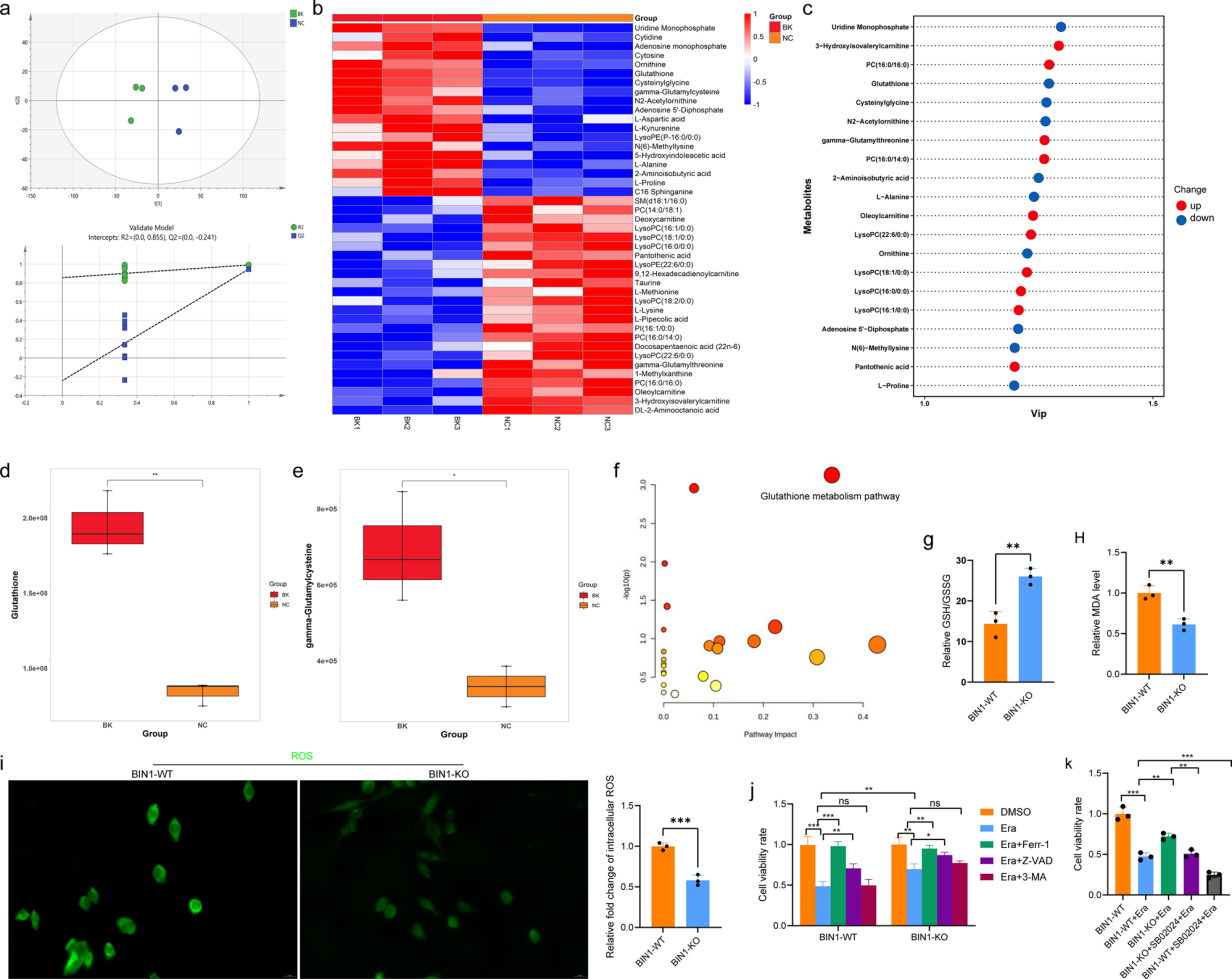
BIN1 Regulates Ferroptosis in NSCLC Cells via the STAT1/GSH Pathway **a**: PLS-DA analysis. **b–e**: Expression levels of metabolites in BIN1-KO cells. **f**: KEGG analysis of differentially expressed metabolites between the BIN1-WT and BIN1-KO cell groups. **g**: GSH/GSSG ratio in BIN1-WT and BIN1-KO cells. **h–i**: Levels of MDA and ROS in BIN1-WT and BIN1-KO cells. **j**: CCK-8 assay used to evaluate the effects of various death inducers and inhibitors on the viability of BIN1-WT and BIN1-KO cells. **k**: The effect of the STAT1 agonist SB02024 on the activity of Erastin-induced BIN1-KO cells analyzed via CCK-8

To further elucidate the biological processes underlying BIN1’s role in NSCLC progression, we performed untargeted metabolomics to identify the critical metabolic pathways associated with BIN1 function. Through PLS-DA analysis, we observed a clear segregation of metabolic profiles between the BIN1-KO and BIN1-WT groups, signifying a significant and consistent metabolic shift resulting from altered BIN1 expression levels (Fig. 6a). The findings revealed that metabolites such as glutathione (GSH) and gamma-glutamylcysteine were markedly upregulated in BIN1 knockout cells (Fig. 6b-e). Moreover, KEGG pathway analysis identified glutathione metabolism in cancer as the most significantly altered pathway, based on the number of differing metabolites and statistical relevance (Fig. 6f). Additionally, we observed an increase in the GSH/GSSG ratio within the BIN1 knockout group (Fig. 6g). In summary, these results demonstrate that BIN1 knockout leads to glutathione accumulation in LLC cells.

The intracellular GSH levels in tumor cells are strongly linked to ferroptosis. To explore the connection between BIN1 and ferroptosis, we performed malondialdehyde (MDA) detection and reactive oxygen species (ROS) measurement. Our findings revealed that MDA and ROS levels were significantly reduced in comparison to the BIN1-WT group (Fig. 6h and i). Subsequently, to further investigate the relationship between BIN1 and ferroptosis, we conducted a CCK-8 assay and analyzed morphological changes in cells under various treatment conditions. As illustrated in Fig. 6j, BIN1 knockout significantly suppressed erastin-induced cell death in LLC cells when compared to the BIN1-WT group. This suppressive effect was reversed by ferrostatin-1 and the apoptosis inhibitor Z-VAD-FMK but remained unaffected by the autophagy inhibitor 3-methyladenine.

To further determine whether BIN1 modulates ferroptosis in NSCLC cells through the STAT1/GSH pathway, we conducted an additional CCK-8 assay (Fig. 6k). The results showed that, compared to the BIN1-WT group, cell viability was significantly lower in the BIN1-WT + Erastin group. Furthermore, compared to the BIN1-WT + Erastin group, cell viability was markedly higher in the BIN1-KO + Erastin group. Notably, compared to the BIN1-KO + Erastin group, cell viability significantly decreased in the BIN1-KO + SB02024 + Erastin group. These observations suggest that BIN1 knockout diminishes the sensitivity of NSCLC cells to ferroptosis inducers, while the STAT1 agonist SB02024 can effectively counteract this effect. These results demonstrate that BIN1 regulates ferroptosis in NSCLC cells through the STAT1/GSH pathway.

### BIN1 modulates the immune microenvironment and progression in NSCLC via the G3BP1/STAT1 axis

**Fig. 7:**
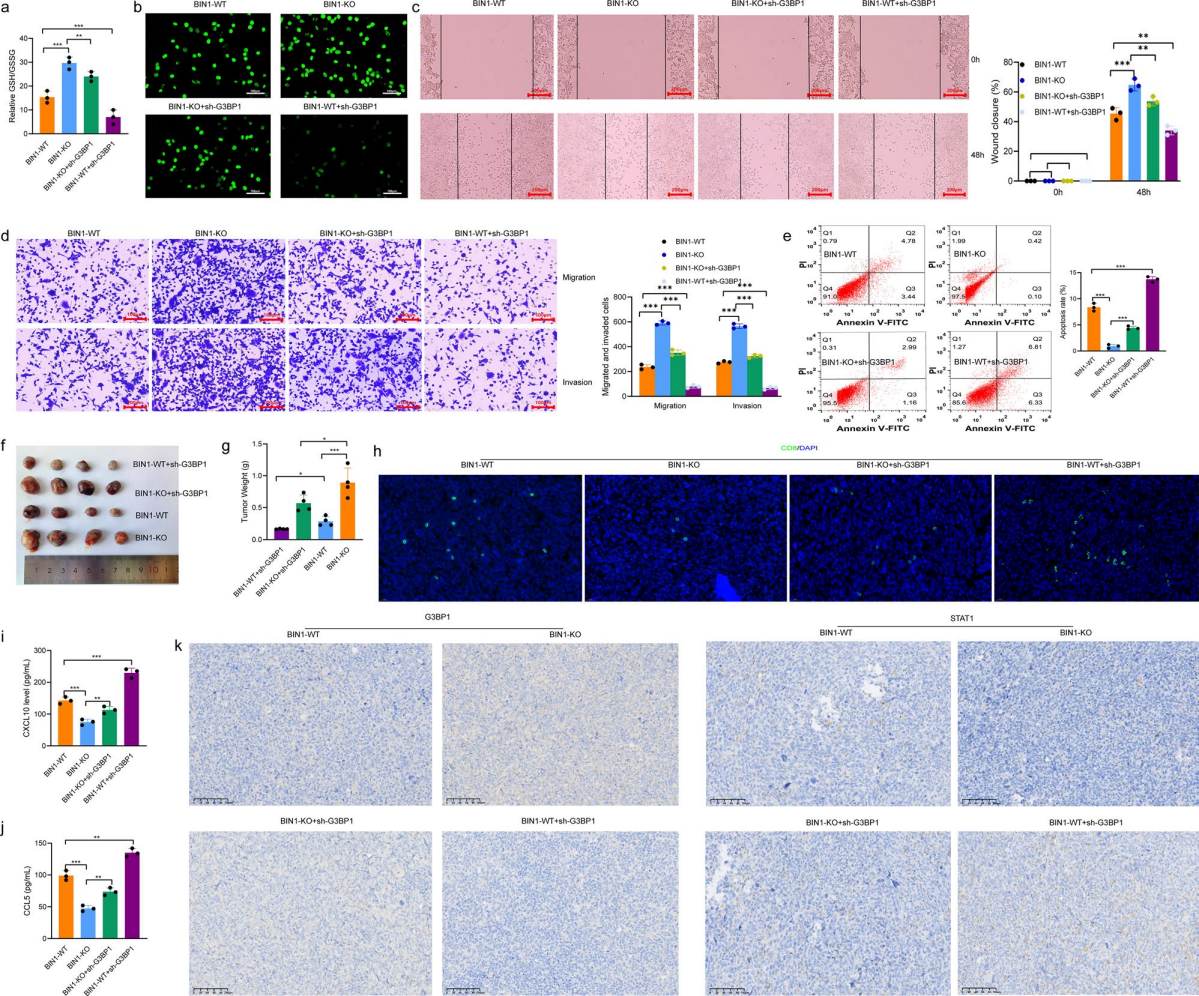
BIN1 modulates the immune microenvironment and progression of NSCLC via the G3BP1/STAT1 axis. **a**: The impact of G3BP1 knockdown on the GSH/GSSG ratio in BIN1-KO cells. **b**: The effect of G3BP1 knockdown on cell proliferation in BIN1-KO cells. **c**: The influence of G3BP1 knockdown on cell migration in BIN1-KO cells. **d**: The role of G3BP1 knockdown in modulating both migration and invasion in BIN1-KO cells. **e**: The effect of G3BP1 knockdown on apoptosis in BIN1-KO cells. **f–g**: The influence of G3BP1 knockdown on the tumorigenic ability of BIN1-KO cells. **h**: The impact of G3BP1 knockdown on CD8^+^ T-cell infiltration in tumors derived from BIN1-KO cells. **i**: The effect of G3BP1 knockdown on CXCL10 expression in tumors derived from BIN1-KO cells. **j**: The effect of G3BP1 knockdown on CCL5 expression in tumors derived from BIN1-KO cells. **k**: The impact of G3BP1 knockdown on Ki-67 and STAT1 expression in tumors derived from BIN1-KO cells

**Fig. 8:**
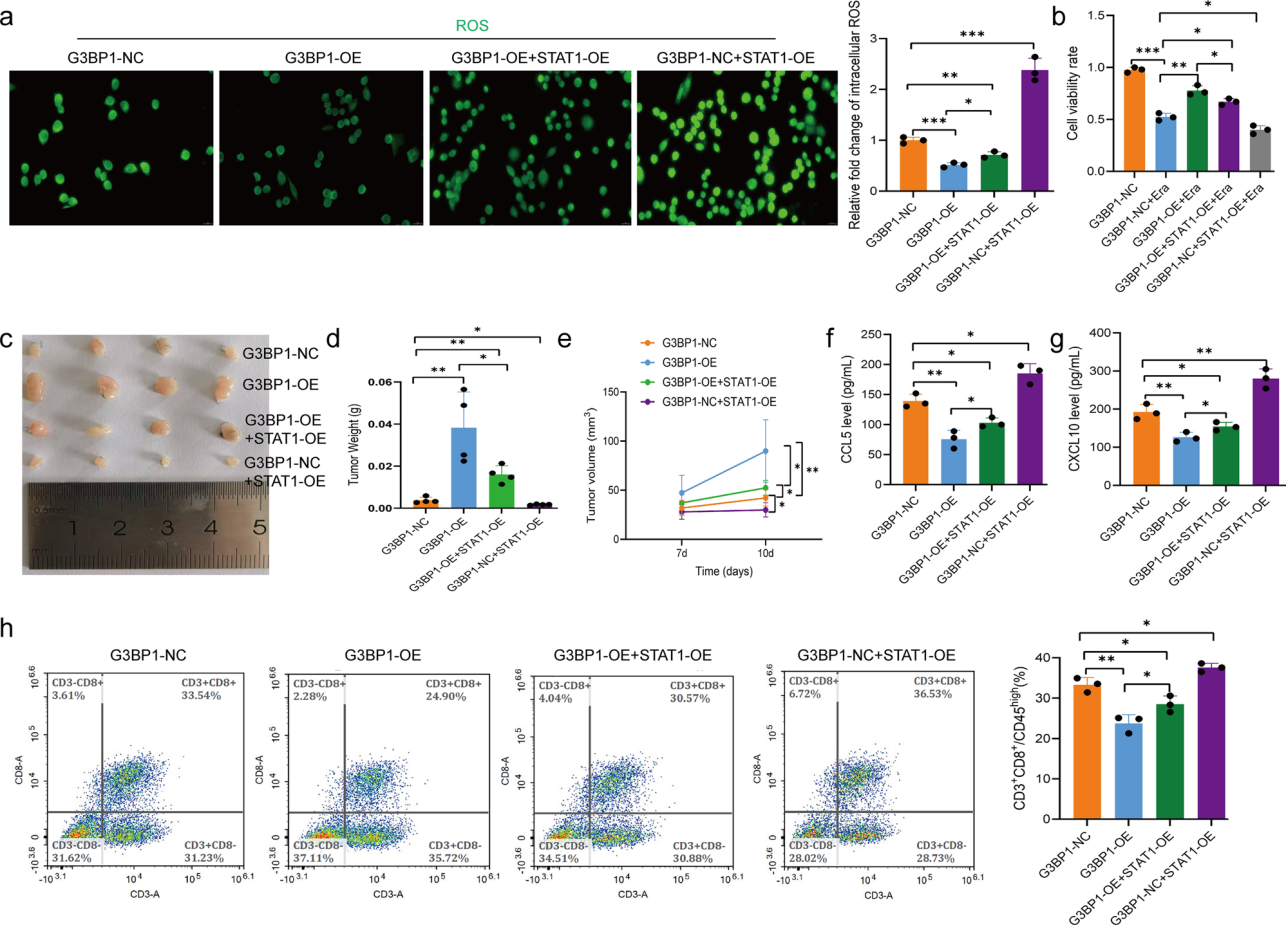
G3BP1 modulates the immune microenvironment and progression of NSCLC via STAT1. **a**: The impact of the STAT1 overexpression on the ROS level of G3BP1-OE cells. **b**: The effect of the STAT1 overexpression on the activity of Erastin-induced G3BP1-OE cells analyzed via CCK-8. **c-e**: The impact of the STAT1 overexpression on the tumorigenic potential of G3BP1-OE cells. **f**: The effect of the STAT1 overexpression on CCL5 expression in tumors derived from G3BP1-OE cells. **g**: The influence of the STAT1 overexpression on CXCL10 expression in tumors derived from G3BP1-OE cells. **h**: The role of the STAT1 overexpression in modulating CD8^+^ T cell infiltration within tumors derived from G3BP1-OE cells

To investigate whether BIN1 promotes the progression of NSCLC through the G3BP1/STAT1 pathway, we developed a BIN1 knockout cell line with reduced G3BP1 expression. Initially, we observed that G3BP1 knockout could mitigate the GSH induction triggered by BIN1 knockout in NSCLC cells (Fig. 7a). Results from the EdU assay revealed that the proliferation of cells in the BIN1-WT + sh-G3BP1 group was significantly lower than that in the BIN1-WT group. Similarly, the proliferation of cells in the BIN1-KO + sh-G3BP1 group was markedly reduced compared to the BIN1-KO group (Fig. 7b). The scratch healing assay demonstrated that the migration repair capacity of cells in the BIN1-WT + sh-G3BP1 group was notably diminished compared to the BIN1-WT group. Likewise, the migration repair ability in the BIN1-KO + sh-G3BP1 group was significantly impaired relative to the BIN1-KO group (Fig. 7c). Moreover, the Transwell migration and invasion assays indicated that the migration and invasion abilities of cells in the BIN1-WT + sh-G3BP1 group were significantly reduced compared to the BIN1-WT group. Similarly, these abilities were markedly decreased in the BIN1-KO + sh-G3BP1 group compared to the BIN1-KO group (Fig. 7d). Apoptosis detection results further revealed that, compared to the BIN1-WT group, the level of apoptosis was significantly upregulated in the BIN1-WT + sh-G3BP1 group. Likewise, apoptosis levels in the BIN1-KO + sh-G3BP1 group were significantly elevated compared to the BIN1-KO group (Fig. 7e). In summary, knockdown of G3BP1 partially counteracts the regulatory effects of BIN1 on the proliferation, migration, invasion, and apoptosis of LLC cells. To further verify that BIN1 mediates its effects via the G3BP1/STAT1 axis, we established LLC cell lines overexpressing both G3BP1 and STAT1 (Supplementary Fig. 5h-i). EDU and Transwell assays revealed that G3BP1 overexpression promoted the proliferation, migration, and invasion of LLC cells, whereas STAT1 overexpression attenuated these effects induced by G3BP1 overexpression (Supplementary Fig. 5e-f). Additionally, overexpression of G3BP1 significantly suppressed ROS levels in LLC cells, whereas STAT1 overexpression partially abrogated this suppressive effect (Fig. 8a). Furthermore, G3BP1 overexpression suppressed Erastin-induced ferroptosis in LLC cells, an effect that was counteracted by STAT1 overexpression (Fig. 8b). These findings suggest that BIN1 regulates the malignant biological behaviors of LLC cells through G3BP1/STAT1 axis.

Importantly, we investigated whether BIN1 reshapes the immune microenvironment of NSCLC via the G3BP1/STAT1 pathway in vivo. Tumor formation assays demonstrated that tumorigenicity in mice from the BIN1-WT + sh-G3BP1 group was markedly reduced compared to the BIN1-WT group. Similarly, tumorigenicity in mice from the BIN1-KO + sh-G3BP1 group was significantly decreased compared to the BIN1-KO group (Fig. 7f, g). Fluorescence tissue staining revealed a substantial increase in the number of CD8-positive T cells within tumor tissues in the BIN1-WT + sh-G3BP1 group, compared to the BIN1-WT group. Likewise, the number of CD8-positive T cells in the BIN1-KO + sh-G3BP1 group was significantly higher than in the BIN1-KO group (Fig. 7h). ELISA results indicated that serum levels of CXCL10 and CCL5 were notably elevated in the BIN1-WT + sh-G3BP1 group compared with the BIN1-WT group. A similar elevation in CXCL10 and CCL5 levels was observed in the BIN1-KO + sh-G3BP1 group compared to the BIN1-KO group (Fig. 7i, j). IHC analysis further demonstrated a significant reduction in the positive rates of G3BP1 expression and upregulation in the positive rates of STAT1 expression within tumor tissues in the BIN1-WT + sh-G3BP1 group compared to the BIN1-WT group. Correspondingly, compared with the BIN1-KO group, the BIN1-KO + sh-G3BP1 group exhibited a significantly reduced positive rate of G3BP1 expression in tumor tissues, whereas the positive rate of STAT1 expression was markedly increased (Fig. 7k). Meanwhile, we observed that G3BP1 overexpression promoted the tumorigenicity of LLC cells and inhibited CD8^+^T cells infiltration (Fig. 8c-h). In contrast, STAT1 overexpression partially attenuated the tumor-promoting effects of G3BP1 overexpression and its suppression of CD8^+^ T cell infiltration (Fig. 8c-h).

In summary, BIN1 regulates STAT1 expression through its interaction with G3BP1. STAT1, in turn, plays a dual role: it modulates CD8^+^ T-cell infiltration by controlling the expression of the chemokines CXCL10 and CCL5, and it affects ferroptosis in NSCLC cells via the GSH pathway.

## Discussion

The effectiveness of immunotherapies in NSCLC largely relies on robust T cell infiltration. However, the mechanisms driving these observations remain inadequately understood. In this study, we propose that the interaction between NSCLC cell-intrinsic BIN1 and CD8^+^ T cells within the TME plays a significant role in NSCLC progression. RNA-seq analysis of BIN1-KO cells and tumors revealed a marked downregulation of STAT1 and its downstream chemokines in both BIN1-KO cells and tumor tissues. The reduced expression of STAT1 not only impairs CD8^+^ T cell infiltration and cytotoxic activity but also facilitates the malignant behavior of NSCLC cells. Mechanistically, IP mass spectrometry and Co-IP analysis demonstrated that BIN1 interacts with G3BP1. The absence of BIN1 enhances the protein stability of G3BP1, which subsequently increases its binding to STAT1, thereby suppressing STAT1 expression. This chain of events ultimately downregulates the expression of CXCL10 and CCL5, leading to diminished CD8^+^ T cell infiltration in tumors. Additionally, our findings reveal that BIN1 influences ferroptosis in NSCLC cells through the BIN1/G3BP1/STAT1/GSH pathway.

Researchers utilized a modified Cre-lox technique to generate BIN1 chimeric mice and documented the incidence of various cancer types. A notable observation was that BIN1 suppresses the prolonged reproductive capacity of aging female mice, while its loss facilitates tumor initiation and progression [24]. Specifically, 50% of BIN1 chimeric mice developed lung cancer, followed by liver cancer [24]. Consistent with prior studies, our results revealed that knocking out BIN1 in tumor cells significantly enhances the development of NSCLC both in vitro and in vivo, underscoring the role of reduced BIN1 expression in driving cancer progression. Intriguingly, the ablation of endogenous BIN1 in tumor cells not only accelerates tumor progression but also diminishes CD8^+^ T cell infiltration and impairs their functionality. This indicates that BIN1 within the TME-not just within tumor cells—plays a critical role in promoting NSCLC progression. Beyond its well-established function in preventing malignant transformation, BIN1 has also been implicated in tumor immune evasion and resistance to chemotherapy [19, 25]. However, the specific relationship between BIN1 and CD8^+^ T cell infiltration remains insufficiently understood. Through RNA-seq analysis, we demonstrated that BIN1 knockout markedly suppresses STAT1 expression in tumor cells and tissues. Subsequent in vitro and in vivo experiments confirmed that the absence of BIN1 promotes the malignant behavior of NSCLC cells by inhibiting STAT1 expression and creates an immunosuppressive microenvironment characterized by reduced CD8^+^ T cell presence. These findings further emphasize the pivotal role of BIN1 in regulating STAT1 expression and its significance in NSCLC progression.

To elucidate the regulatory network of BIN1 in the remodeling and progression of the immune microenvironment in NSCLC, we performed IP-MS experiments. Furthermore, by employing molecular docking and Co-IP assays, we validated that G3BP1 serves as a downstream interacting protein of BIN1. G3BP1, also referred to as a stress granule component factor, exhibits ATP-dependent helicase activity and was the first Ras-GTPase activating protein (GAP) binding protein identified through Co-IP. It plays a critical role in Ras pathway signal transduction and is highly conserved across eukaryotic evolution [26]. Research has demonstrated that G3BP1 significantly enhances cGAS enzymatic activity, thereby promoting the activation of the cGAS-mediated type I interferon signaling pathway and contributing to innate immune regulation [27]. In bladder cancer, G3BP1 collaborates with SLU7 to activate the PI3K/AKT pathway, leading to reduced MHC-I expression and facilitating immune escape. Additionally, G3BP1 inhibitors can suppress the PI3K/AKT signaling pathway, restore MHC-I expression, and enhance the efficacy of PD-1 monoclonal antibody therapy [28]. Our research demonstrates that BIN1 interacts with G3BP1, and the knockout of BIN1 significantly enhances G3BP1 stability. Furthermore, we demonstrated that BIN1 loss inhibits CD8^+^ T cell infiltration by stabilizing G3BP1, which in turn induces the establishment of an immunosuppressive microenvironment and facilitates tumor progression.

Further analysis using protein interaction prediction tools and Co-IP experiments revealed that G3BP1 binds to STAT1, resulting in reduced STAT1 expression. STAT1, the first member of the STAT family, plays a crucial role in interferon (IFN) signaling. The bulk of the evidence indicates that the activation of STAT1 exerts tumor-suppressive effects in cancer cells. Studies demonstrate that vitamin C directly modifies lysine residues, leading to a novel post-translational modification termed “lysine vitcylation”. Furthermore, it is revealed that this process strengthens the interferon signaling pathway by stabilizing STAT1 phosphorylation, thereby activating the anti-tumor immune response [29]. The study conducted by Zheng et al. revealed that the inactivation of SETD2 leads to the activation of the STAT1 signaling pathway through the reduction of NR2F1 transcription. This process subsequently enhances the expression of chemokines and PD-1, improves antigen presentation, and ultimately strengthens the immune response [30]. Nevertheless, evidence from specific experimental and clinical studies suggests that STAT1 can display tumor-promoting activities under defined conditions [31–32]. In certain malignant phenotypes, STAT1 may function as either an oncoprotein or a tumor suppressor within the same cell type, depending on the unique genetic background. Our findings demonstrate that STAT1 overexpression partially offsets the suppressive effects of BIN1 knockout and G3BP1 overexpression on the immune microenvironment in NSCLC. These findings confirm that the knockout of BIN1 increases both the stability of G3BP1 and its binding affinity for STAT1, thereby suppressing the expression of CXCL10 and CCL5 via decreased STAT1 levels, highlighting potential therapeutic applications for NSCLC patients. However, further studies are necessary to fully elucidate the mechanisms by which BIN1 regulates the proliferation, migration, and invasion of NSCLC.

To further clarify the molecular mechanisms through which BIN1 influences the progression of NSCLC, we performed metabolomic analyses on BIN1-WT and BIN1-KO cells. Our results revealed that GSH levels were significantly upregulated in BIN1-knockout cells. GSH, a tripeptide comprising glutamic acid, cysteine, and glycine, functions as an antioxidant and serves as the substrate for glutathione peroxidase 4 (GPX4), which converts it into oxidized glutathione (GSSG) [33–37]. Elevated GSH levels are known to inhibit ferroptosis [38–42]. To investigate whether the GSH accumulation induced by BIN1 knockout suppresses ferroptosis, we assessed the GSH/GSSG ratio, ROS levels, and MDA content in each cell group. The findings demonstrated that BIN1 knockout led to a significant increase in the GSH/GSSG ratio while concurrently reducing ROS levels and MDA content. Moreover, CCK-8 assays showed that ferroptosis inhibitors were able to counteract erastin-induced cell death in both BIN1-WT and BIN1-KO cells. Importantly, BIN1 knockout diminished sensitivity to ferroptosis inducers by inhibiting STAT1 activity, thereby facilitating NSCLC progression. In summary, our results highlight that BIN1 knockout elevates GSH levels in NSCLC cells via the G3BP1/STAT1 pathway, effectively suppressing ferroptosis and driving the malignant progression of NSCLC.

## Conclusion

Our study underscores the pivotal role of the BIN1-G3BP1-STAT1 axis in driving immune suppression and facilitating the progression of NSCLC. These findings emphasize the therapeutic potential of targeting BIN1, G3BP1, and STAT1, especially in the context of NSCLC treatment. However, further research is essential to comprehensively evaluate the prognostic and therapeutic implications of these targets.

## Electronic supplementary material

Below is the link to the electronic supplementary material.

Supplementary Material 1

Supplementary Material 2: Supplementary Fig. 1 The relationship between BI1N1 expression and the clinicopathological characteristics of lung cancer patients a: The expression levels of BIN1 protein in different pathological types of lung cancer. b: The expression level of BIN1 protein in lung cancer patients of different genders. c: The expression level of BIN1 protein in lung cancer patients of different ages. d: The relationship between BIN1 expression levels and the survival period of patients with lung adenocarcinoma. e: Label for BIN1 expression detection in tissue microarray. f: Label for CD8 expression detection in tissue microarray. g-h: Kaplan-Meier survival analysis was performed to evaluate the association between BIN1 expression levels and the prognosis of NSCLC patients across different clinical stages.

Supplementary Material 3: Supplementary Fig. 2 The effect of BIN1 knockout on the proliferation, migration, invasion and apoptosis of lung cancer cells a: Information on the vector used for BIN knockout. b: Sanger sequencing was used to detect the knockout efficiency of BIN1. c: Sequence comparison of BIN1 knockout. d: Western blot was used to detect the knockout efficiency of BIN1 in LLC cells. e: The effect of BIN1 knockout on the proliferation ability of LLC cells. f: The effect of BIN1 knockout on the invasion ability of LLC cells. g-h: The effect of BIN1 knockout on the migration and invasion ability of LLC cells. i: The effect of BIN1 knockout on the apoptosis of LLC cells.

Supplementary Material 4: Supplementary Fig. 3 The regulatory effect of BIN1 knockout on the immune microenvironment of NSCLC a: Flow cytometry mass spectrometry was used to detect the changes in immune cell subsets in the spleens of BIN1 knockout and control mice. b: Flow cytometry mass spectrometry was used to detect the changes in immune cell subsets in the lymph nodes of BIN1 knockout and control mice. c: Flow cytometry was used to detect the effect of BIN1 knockout on the proportion of macrophages in mouse tumor tissues. d: Flow cytometry was used to detect the effect of BIN1 knockout on the proportion of MDSCs in mouse tumor tissues. e: Flow cytometry was used to detect the effect of BIN1 knockout on the proportion of monocytes in mouse tumor tissues. f: Flow cytometry was used to detect the effect of BIN1 knockout on the proportion of neutrophils in mouse tumor tissues. g: Flow cytometry was used to detect the effect of BIN1 knockout on the proportion of NKT cells in mouse tumor tissues.

Supplementary Material 5: Supplementary Fig. 4 Analysis of the Expression and Clinical Significance of G3BP1 and STAT1 in NSCLC a: Molecular docking was used to explore the binding ability and sites of G3BP1 and STAT1. b-c: Database analysis was conducted to examine the expression levels of G3BP1 and STAT1 in NSCLC. d-e: Database analysis was carried out to determine the expression levels of G3BP1 and STAT1 in patients with different clinical stages. f: Kaplan-Meier analysis was applied to assess the relationship between G3BP1 expression and NSCLC survival. g: Kaplan-Meier analysis was used to evaluate the association between STAT1 expression and NSCLC survival. h-i: The overexpression efficiency of G3BP1-OE and STAT1-OE was respectively detected by RT-qPCR.

Supplementary Material 6: Supplementary Fig. 5 BIN1 affects the proliferation, migration and invasion of NSCLC cells through STAT1. a: CCK-8 assay was used to determine the IC50 values of BIN1-WT and BIN1-KO cells under the intervention of STAT1 agonist SB02024. b: EdU assay was conducted to detect the changes in cell proliferation ability among different groups. c: Scratch wound healing assay was performed to examine the changes in cell migration ability among different groups. d: Transwell assay was carried out to assess the changes in cell migration and invasion ability among different groups. e: EdU assay was conducted to detect the changes in cell proliferation ability among different groups. f: Transwell assay was carried out to assess the changes in cell migration and invasion ability among different groups.

## Data Availability

No datasets were generated or analysed during the current study.
