# Supplementary material for: Tumor cell-intrinsic BIN1 deficiency promotes the immunosuppression and impedes ferroptosis of non-small cell lung cancer via G3BP1-mediated degradation of STAT1
Source: J Exp Clin Cancer Res. 2025 May 9;44:141. doi: 10.1186/s13046-025-03404-9 (PMC12063428; doi:10.1186/s13046-025-03404-9)
Supplement: Supplementary file 1 — Supplementary Material 1 [file 13046_2025_3404_MOESM1_ESM.docx]

Table S1 KEGG enrichment analysis

| **Pathway Name** | **-log(p)** | **Impact** |
| --- | --- | --- |
| Glutathione metabolism | 3.126 | 0.33736 |
| Arginine biosynthesis | 2.9548 | 0.06091 |
| Lysine degradation | 1.9793 | 0.00204 |
| Pantothenate and CoA biosynthesis | 1.4218 | 0.0068 |
| Alanine, aspartate and glutamate metabolism | 1.156 | 0.22356 |
| Linoleic acid metabolism | 1.1178 | 0 |
| Glycerophospholipid metabolism | 0.96698 | 0.11182 |
| Arginine and proline metabolism | 0.96698 | 0.18139 |
| Taurine and hypotaurine metabolism | 0.92339 | 0.42857 |
| Pyrimidine metabolism | 0.90874 | 0.09117 |
| Tryptophan metabolism | 0.87288 | 0.10807 |
| Biotin metabolism | 0.83294 | 0 |
| Caffeine metabolism | 0.7602 | 0.30769 |
| alpha-Linolenic acid metabolism | 0.72865 | 0 |
| Nicotinate and nicotinamide metabolism | 0.6729 | 0 |
| Histidine metabolism | 0.64805 | 0 |
| Selenocompound metabolism | 0.56382 | 0 |
| beta-Alanine metabolism | 0.54578 | 0 |
| Purine metabolism | 0.51152 | 0.07914 |
| Sphingolipid metabolism | 0.39705 | 0 |
| Cysteine and methionine metabolism | 0.38675 | 0.10446 |
| Arachidonic acid metabolism | 0.30204 | 0 |
| Primary bile acid biosynthesis | 0.28168 | 0.02239 |
