# Supplementary figures and images for: Tumor cell-intrinsic BIN1 deficiency promotes the immunosuppression and impedes ferroptosis of non-small cell lung cancer via G3BP1-mediated degradation of STAT1
Source: J Exp Clin Cancer Res. 2025 May 9;44:141. doi: 10.1186/s13046-025-03404-9 (PMC12063428; doi:10.1186/s13046-025-03404-9)

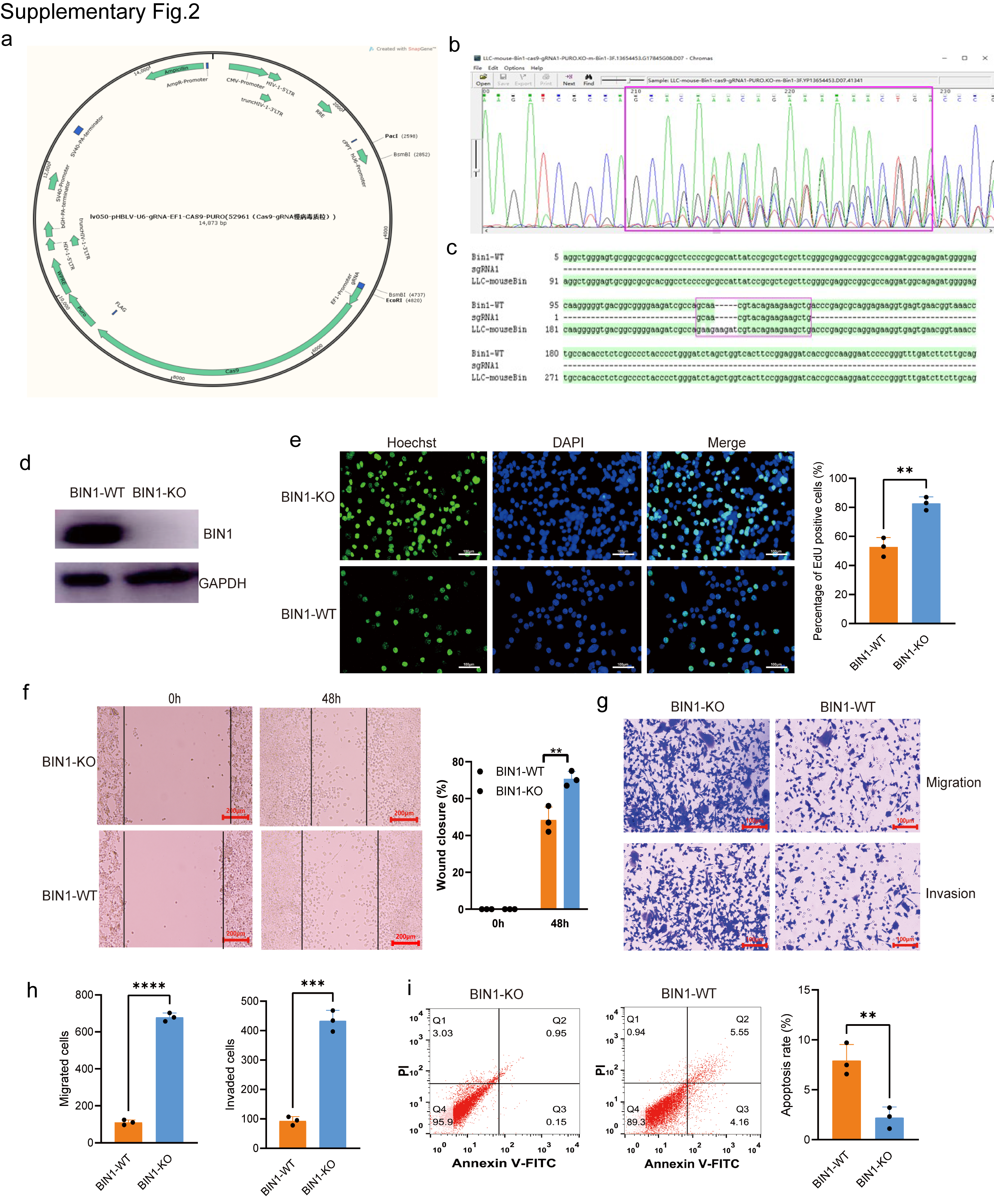

Supplement: Supplementary file 3 — Supplementary Material 3: Supplementary Fig. 2 The effect of BIN1 knockout on the proliferation, migration, invasion and apoptosis of lung cancer cells a: Information on the vector used for BIN knockout. b: Sanger sequencing was used to detect the knockout efficiency of BIN1. c: Sequence comparison of BIN1 knockout. d: Western blot was used to detect the knockout efficiency of BIN1 in LLC cells. e: The effect of BIN1 knockout on the proliferation ability of LLC cells. f: The effect of BIN1 knockout on the invasion ability of LLC cells. g-h: The effect of BIN1 knockout on the migration and invasion ability of LLC cells. i: The effect of BIN1 knockout on the apoptosis of LLC cells. [file 13046_2025_3404_MOESM3_ESM.tif]
